# The potential role of patent and proprietary medicine vendors’ associations in improving the quality of services in Nigeria’s drug shops

**DOI:** 10.1186/s12913-020-05379-z

**Published:** 2020-06-22

**Authors:** Abisoye S. Oyeyemi, Oladimeji Oladepo, Adedayo O. Adeyemi, Musibau A. Titiloye, Sarah M. Burnett, Iorwakwagh Apera

**Affiliations:** 1 Department of Community Medicine, Niger Delta University, Wilberforce Island, Bayelsa State Nigeria; 2 Department of Health Promotion and Education, Faculty of Public Health, College of Medicine, University of Ibadan, Ibadan, Nigeria; 3 Centre for Infectious Diseases Research and Evaluation, Abuja, Nigeria; 4 Accordia Global Health Foundation, now Africare, Washington, DC USA; 5 West African Infectious Diseases Institute, Abuja, Nigeria

**Keywords:** Association, iCCM, NAPPMED, Regulatory agencies, Patent and proprietary medicine vendors

## Abstract

**Background:**

Patent and Proprietary Medicine Vendors (PPMVs) play a major role in Nigeria’s health care delivery but regulation and monitoring of their practice needs appreciable improvement to ensure they deliver quality services. Most PPMVs belong to associations which may be useful in improving their regulation. However, little is known about how the PPMV associations function and how they can partner with relevant regulatory agencies to ensure members’ compliance and observance of good practice. This study sought to describe the PPMV associations’ structure and operations and the regulatory environment in which PPMVs function. With this information we explore ways in which the associations could help improve the coverage of Nigeria’s population with basic quality health care services.

**Methods:**

A mixed methods study was conducted across four rural local government areas (LGAs) (districts) in two Nigerian states of Bayelsa and Oyo. The study comprises a quantitative data collection of 160 randomly selected PPMVs and their shops, eight PPMV focus group discussions, in-depth interviews with 26 PPMV association executives and eight regulatory agency representatives overseeing PPMVs’ practice.

**Results:**

The majority of the PPMVs in the four LGAs belonged to the local chapters of National Association of Patent and Proprietary Medicine Dealers (NAPPMED). The associations were led by executive members and had regular monthly meetings. NAPPMED monitored members’ activities, provided professional and social support, and offered protection from regulatory agencies. More than 80% of PPMVs received at least one monitoring visit in the previous 6 months and local NAPPMED was the organization that monitored PPMVs the most, having visited 68.8% of respondents. The three major regulators, who reached 30.0–36.3% of PPMVs reported lack of human and financial resources as the main challenge they faced in regulation.

**Conclusions:**

Quality services at drug shops would benefit from stronger monitoring and regulation. The PPMV associations already play a role in monitoring their members. Regulatory agencies and other organizations could partner with the PPMV associations to strengthen the regulatory environment and expand access to basic quality health services at PPMV shops in Nigeria.

## Background

Universal health coverage (UHC) entails providing access to quality health services to all people wherever they live [1]. Achieving UHC is a priority for many countries including Nigeria and it involves harnessing human and material resources available in both the public and private sectors [2]. In many resource-poor countries, including Nigeria, the private sector plays a major role in health care delivery, providing health care services to a significant proportion of the population. For example, the 2018 Nigeria Demographic and Health Survey (NDHS) shows that the private sector provided modern contraceptives for 41% of users and was the place of institutional delivery for 33% of women [3]. For childhood illnesses, treatment was sought for 62.3% of under five children with fever, 63.5% with diarrhoea, and 50.5% with acute respiratory infection in the private sector.

Notable among the private sector players in Nigeria are drug shop owners, known as patent and proprietary medicine vendors (PPMVs), who are “persons without formal pharmacy training selling orthodox pharmaceutical products on a retail basis for profit” [4]. The number of PPMVs was estimated to be 200,000 in 2005, roughly 100 times greater than the number of registered pharmacies, and nearly four times the number of physicians [5]. A recent study also reported a drug shop density comparable to or higher than formal health facilities in many parts of the country [6]. PPMVs act as the first point of care for majority of the Nigerian population, providing services for malaria, diarrhoea, pneumonia, and family planning, and caring for both children and adults [3, 7–12] especially in the rural areas [13, 14]. The PPMVs also have many qualities that appeal to members of the community such as long opening hours, hospitability, and more consistent availability than public facilities that frequently experience service interruptions [15, 16]. Given their widespread presence, well-regulated and well-trained PPMVs could boost Nigeria’s progress towards UHC by being more involved in promising health interventions such as integrated community case management (iCCM) [17].

PPMVs practice is primarily regulated by the Pharmacists Council of Nigeria (PCN) [18, 19]. There are various concerns however, about their adherence to rules and regulations guiding their practice [6, 11, 12, 18, 20, 21]. There are also concerns about their knowledge of the conditions they manage and the quality of service they render [21–26]. These findings may suggest poor regulation and monitoring in the context of a weak health system.

PPMVs are organized into local associations in many parts of the country and these associations have unique influence among PPMV populations. Previous works suggest that PPMV associations could be a powerful partner in ensuring that PPMVs adhere to extant regulatory requirements and deliver high quality services in interventions aimed at improving PPMV treatment practices [27, 28]. Several studies and intervention programmes have focused on individual PPMVs but only a few have actively engaged with the PPMV associations. Little is known about the structure and operations of PPMV associations and the regulatory environment in which PPMVs operate.

In this study we describe PPMV associations and their operations in the rural areas of Bayelsa and Oyo states of Nigeria, the existing regulatory environment including monitoring and supervision of PPMVs, and the implications of these findings for improving the quality of services provided by the PPMVs. This paper emanates from the Strengthening Patent and Proprietary Medicine Vendors’ Associations in Nigeria for Improved Malaria Management (SPANIMM) operations research project. SPANIMM aimed to improve PPMVs’ malaria case management. In the first phase of this research, the objectives were to: a) determine PPMVs’ current malaria case management knowledge and practices and b) describe the structure and operations of the PPMV associations and the PPMV regulatory environment to inform strategies that could improve the quality of malaria care at PPMV shops. A paper reporting the first objective has already been published [26]. This paper focuses on the second objective to describe PPMV’s association and regulatory environment.

## Methods

### Study design

A mixed methods approach involving cross-sectional study design and qualitative study was used to assess and to inform the design of an intervention to improve PPMV treatment practices. The quantitative part involved administration of structured questionnaire (Additional file 1) to PPMVs on shop owner characteristics and involvement with the PPMV association and regulatory agencies. Focus group discussions (FGDs) with PPMVs were done using FGD guide (Additional file 2) to further describe their interactions with the PPMV association and regulatory agencies. Semi-structured interviews with PPMV association executives were conducted with interview guides (Additional file 3) and key informant interviews with regulatory agency representatives were conducted (Additional file 4) to describe the organizations’ structures, key functions, roles and experiences regulating PPMVs.

### Study population

The study was conducted in two purposively selected states in Nigeria, Oyo and Bayelsa. The states represented different geographic and linguistic-ethnic regions in southern Nigeria. From each state, two rural local government areas (LGAs) were selected. Of the 33 LGAs in Oyo state, 12 are rural. From these 12, Ibarapa East and Kajola were randomly selected. Of the eight LGAs in Bayelsa state, seven are rural. From these seven, the LGAs were further stratified by landed and riverine, with one landed rural LGA (out of three), Ogbia, and one riverine LGA (out of four), Brass, randomly selected. Within each LGA, the key study populations were PPMVs, the local PPMV associations, and regulatory agencies. Within each LGA, two PPMV FGDs were held, one each for male and female PPMV association members.

### Sample size and sampling technique

The sample size for the quantitative part of the operations research was 160 (40 PPMVs per LGA). The estimation has been explained in a previous publication [26]. Within each LGA, national association of patent and proprietary medicine dealers (NAPPMED) executives provided a list of NAPPMED PPMVs. The list contained members registered and not registered with PCN. Simple random sampling was used to select 160 PPMVs from the list of 636 PPMVs to participate in the survey; 137, 350, 51 and 98 from Ibarapa East, Kajola, Brass and Ogbia, respectively. None of the selected PPMVs declined participation.

For the PPMV focus group discussions (FGD), PPMV Association members were approached and those willing to participate were recruited and gathered at an agreed place in the respective LGA. Each FGD had 8–12 members per group. For the qualitative interviews, all LGA PPMV association executives were invited for in-depth interviews, in addition to one state-level PPMV Association executive from each state. Representatives from each of the following regulatory agencies within each state were invited for interviews: Pharmacists Council of Nigeria (PCN), National Agency for Food and Drug Administration and Control (NAFDAC), National Drug Law Enforcement Agency (NDLEA), State Malaria Control Programme (SMCP), and State Ministry of Health (SMOH).

### Data collection

Data collection took place June–August 2013. All data collection tools were translated and administered in the local languages (Yoruba and Pidgin), with the exception of the regulatory agency key informant interviews, which were done in English. Data collection teams were trained over 5 days, training included tool pre-testing and modification prior to data collection.

### Data analysis

Data were collected on paper and cleaned and coded prior to data entry using SPSS for Windows (Version 21.0, IBM Corp., Armonk, NY). SPSS and Stata (Version 11.0 StataCorp, College Station, TX) were used for data analysis. Descriptive statistics with mean, standard deviation, median, and range were generated. English transcripts of FGDs, semi-structured interviews and regulator key informant interviews were entered and edited in Microsoft Word and analyzed using Atlas.ti (Version 7.0; Scientific Software Development GmbH, Berlin, Germany). Based on the participants’ responses to the interview guides, the authors identified key themes and illustrative expressions were extracted.

## Results

A total of 28 PPMV association executives, six to seven within each LGA and the state representative, and representatives from four regulatory agencies in each state were interviewed. During the study it was discovered that the Ogbia LGA PPMV Association had split into three sub–units. The executives from the largest of the three sub-units, with 40 members, were selected for the interviews. Eight focus group discussions were held with 8 to 12 PPMVs in each of the study LGAs. All 160 selected PPMVs participated in the quantitative (shop) survey. Table 1 gives an overview of the data collected during the study.

**Table 1:** Overview of Data Collected

Data Collection Tool	Oyo			Bayelsa			Total
	Ibarapa East	Kajola	State	Brass	Ogbia	State	
**Qualitative**							
PPMV Association Semi-Structured Interview	6	7	1	7	6	1	**28**
Regulator Key Informant Interviews	–	–	4	–	–	4	**8**
PPMV Focus Group Discussions	2	2	–	2	2	–	**8**
*Sub-total*	*8*	*9*	*5*	*9*	*8*	*5*	***44***
**Quantitative**							
PPMV Knowledge Survey with Drug Audit	40	40	–	40	40	–	**160**

### PPMV association structure, administration, and communication

The four LGA PPMV Associations were all local affiliates of the National Association of Patent and Proprietary Medicine Dealers (NAPPMED), a national PPMV association, and had been in operation for 12–41 years (Table 2). Although no formal mapping was done as part of this research study, PPMV association executives estimated that their associations included about 90% of the PPMVs in their LGAs, with the exception of Ogbia, where the executives estimated that their association has over 50% coverage of PPMVs in the area. These LGA NAPPMED branches report to the state NAPPMED, and the state associations report to the national NAPPMED. The LGA NAPPMED associations each met on a monthly basis and had between 51 and 350 members. None of the associations had an office; they usually had their meetings in a member’s house/shop or rented hall. The PPMV Associations communicated with their members mainly through the routine association meetings. They also communicated through phone calls and text messages on their personal phones and in-person shop visits. The main information executives reported sharing with their members include information about upcoming meetings, business and training opportunities, Ministry of Health guidelines, list of approved drugs, information and educational materials and information on upcoming visits from regulators.

**Table 2:** PPMV Association and PPMV Members Description

	Oyo			Bayelsa			Total N (%)
	Ibarapa East N (%)	Kajola N (%)	Total N (%)	Brass N (%)	Ogbia N (%)	Total N (%)	
Total LGA Population^a^	118,226	200,997	319,223	184,127	179,606	363,733	682,956
**LGA PPMV Association**							
Years of Operation	25	34		41	12^b^		
Frequency of Meetings	Monthly	Monthly	–	Monthly	Monthly^b^	–	–
Total PPMV Association Members	137	350	487	51	98	149	636
**PPMVs**	40	40	80	40	40	80	160
Sex: Male	9(22.5)	7 (17.5)	16 (20.0)	21 (52.5)	25 (62.5)	46 (57.5)	62 (38.8)
Age	31.58 ± 6.8 (24–65)	36.33 ± 10.4 (22–66)	33.95 ± 9.071 (22–66)	40.0 ± 12.3 (21–56)	35.5 ± 9.2 (23–67)	37.8 ± 11.0 (21–67)	35.94 ± 10.3 (21–67)
Completed Senior Secondary or more	40(100.0)	30(75.0)	70(87.5)	35(87.5)	38(95.0)	73(91.3)	143 (89.4)
Completed PPMV apprenticeship	40 (100.0)	38 (95.0)	78 (97.5)	26 (65.0)	28 (70.0)	54 (67.5)	132 (82.5)
Completed any Health Professional Education Programme^c^	4 (10.0)	6 (15.0)	10 (12.5)	17 (42.5)	13 (32.5)	30 (37.5)	40 (25.0)
Nursing/Midwifery	0	0	0	0	1 (2.5)	1 (1.25)	1 (0.6)
General Nursing	0	1(2.5)	1 (1.25)	2 (5.0)	2 (5.0)	4 (5.0)	5 (3.1)
Community Health Officer	0	1(2.5)	1 (1.25)	5 (12.5)	4 (10.0)	9 (11.3)	10 (6.3)
Community Health Extension Worker	1(2.5)	1(2.5)	2 (5.0)	2 (5.0)	2 (5.0)	4 (5.0)	6 (3.8)
Auxiliary Nursing	3 (7.5)	3 (7.5)	6 (7.5)	6 (15.0)	3 (7.5)	9 (11.3)	15 (9.4)
Environmental Health Officer	0	0	0	2 (5.0)	1 (2.5)	3 (3.8)	3 (1.9)
Registered with Pharmacists Council of Nigeria (PCN)	32 (80.0)	21 (52.5)	53 (66.3)	11 (27.5)	9 (22.5)	20 (25.0)	73 (45.6)
Of those registered, currently licensed by PCN	18 (56.3)	11 (52.4)	29 (54.7)	5 (45.5)	2 (22.2)	7 (35.0)	36 (49.3)
Very Active/Active in PPMV Association	39 (97.5)	36 (90.0)	75(93.8)	32 (80.0)	31 (77.5)	63 (78.8)	138 (86.3)
Attendance at PPMV Association Meetings	40 (100.)	40 (100.)	80(100.)	39 (97.5)	38 (95.0)	77 (96.3)	157 (98.1)
Paid full PPMV Association Dues in 2012	38 (95.0)	38 (95.0)	76(95.0)	37 (92.5)	33 (82.5)	70(87.5)	146 (91.3)

^a^Total LGA Population from Federal Government Official Gazette. *Legal Notice and Publication of Details of the Breakdown of the National and State Provisional Totals of 2006 Census.* 24(94).15 May 2007

^b^Factors apply only to the Kolo Creek sub-unit of the PPMV Association

^c^ These are Nurses, Auxiliary nurses, Community Health Officers, Community Health Extension Workers and Environmental Health Officers

### Membership and registration with PPMV association

Requirements for membership of the PPMV association were similar across the LGAs. In all four LGAs, members must have completed a PPMV apprenticeship programme, own a PPMV shop and be able to pay the PPMV association dues. In Oyo State LGAs, members must also be 18 years of age, while in the two Bayelsa State LGAs members must have a minimum of senior secondary school education. Of the 160 PPMVs surveyed across the four LGAs, all were 21 years of age or older [fulfilling the minimum age required to be a PPMV by PCN [19]], 143 (89.4%) had a secondary education or higher, and 132 (82.5%) had completed a PPMV apprenticeship (Table 2). In addition, 40 (25.0%) had completed some form of health professional education programme, many more in Bayelsa than in Oyo LGAs (Table 2). In each LGA, PPMVs were expected to pay an initial registration fee followed by annual dues which were paid on a monthly basis during the regular association meetings. While the fees varied widely, even within the LGAs, overall, the fees were much higher in Bayelsa LGAs than in Oyo LGAs. The registration fee ranged from ₦5000 ($31.8) to ₦500,000 ($3184) and the annual dues from ₦1200 ($7.6) to ₦6000 ($38.2), the latter being usually paid as monthly instalments during the monthly meeting. Overall, 138 (86.3%) of PPMV association members sampled reported being either very active or active in their association. As evidence of this level of activity, nearly all of the PPMVs (157, 98.1%) across all four LGAs reported regular attendance at LGA association meetings and paid their full annual dues in the previous year.

### Registration with pharmacists Council of Nigeria (PCN)

All PPMVs are required by Nigerian law to register with the PCN and renew their license annually. Despite these requirements, less than a half (73/160, 45.6%) of PPMVs were registered with PCN, and, of those registered, 36 (49.3%) had an annual license (Table 2). Overall, only 36 (22.5%) of the surveyed PPMVs had both a PCN registration and current license. Registration (66.3% vs. 25.0%) and licensing (54.7% vs. 35.0%) were higher in Oyo than in Bayelsa.

### PPMV association management and functions

The LGA PPMV associations are governed by a body of 6–8 executives elected for a 2–5 year tenure depending on the position and the LGA. The executive positions are voluntary and unpaid and executives reported working about 3–10 h per week on average. As reported by PPMV Association executives the main activities of the association are monitoring members’ activities, such as ensuring that members stock and sell appropriate drugs; ensuring price regulation among members; discouraging sale of counterfeit drugs; social support and welfare benefits through cash/in-kind assistance for members in times of hardship, such as the death of a family member; support in obtaining PCN registration and licensing, legal services and professional development through access to training seminars and updates on new drug regimens. To support these activities, three of the four LGAs have sub-committees led by board members, such as social committees, welfare committees, and education and monitoring task forces.

## Box 1 Key Functions of PPMV Associations

**Table:** 

• Monitoring and ensuring that members stock and sell appropriate drugs; ensuring price regulation among members; discouraging sale of counterfeit drugs.	
• Social support and welfare benefits through cash/in-kind assistance for members in times of hardship, such as the death of a family member;	
• Support in obtaining PCN registration and licensing and legal services	
• Professional development through access to training seminars and updates on new drug regimens	

During the focus group discussions, PPMVs reported that the association supports its members in times of need, acting as a social insurance organization through the social and welfare committees. According to PPMVs from the LGAs in Oyo State:

*“When we have members that have financial challenges, the association can also assist such a person to continue the business. Also, when a member is sick, we normally contributed money for such a person so that he will feel the impact of the association. More so, one major support we normally received from the association is the contributions we do and when we contributed such huge amount of money we assist ourselves to support our business.” – Kajola PPMV, Oyo State*

While not reported by PPMV association executives, members reported that a key service provided by the association and a main reason they joined was protection from regulatory agencies:

*“We join the association for self-defense against the regulatory bodies.”– PPMV, Bayelsa*

### Role of PPMV associations in monitoring and regulating members

All of the PPMV associations reported a role in regulating and monitoring members’ behaviour and three of the four LGAs had a monitoring task force. The main responsibilities of the monitoring task force, as reported by PPMV association executives, are to encourage PPMVs who are not currently members to join the association and to monitor drug stocks. The extent of monitoring varied with frequencies of every 2 weeks, once a month, quarterly, or yearly depending on the LGA.

PPMV Association executives reported that when expired, counterfeit or banned drugs are found in a PPMV’s shop during monitoring visits the drugs are confiscated or burned, the PPMV is fined, and the association may also lock up the PPMV’s shop until the fine is paid or reparations are made. Twelve (7.5%) of the 160 PPMVs surveyed reported being sanctioned by their association. Reasons for sanctions included operating without the knowledge of the association, refusing to pay dues, being late or absent from association meetings and “graduating” an apprentice without informing the PPMV Association. Only one PPMV reported a sanction related to drug stocks - the PPMV was sanctioned for selling drugs at a high price. None of the sanctions were for poor quality of care.

### Training for members

All four of the LGA PPMV Associations reported that they supported regulatory bodies, State Ministry of Health (SMOH), and non-governmental organizations (NGOs) in offering training to their members. In Brass, the executives reported sometimes using the association funds to pay for transportation to training courses. Importantly, the feeling was that any training provided by other organizations should be provided free of charge. In addition to these external trainings, executives in Ibarapa East and Kajola reported proactively scheduled internal trainings for their members. In Ibarapa East they reported that these trainings were held on a quarterly basis.

### Overview of PPMV regulatory environment

Three major organizations were found to monitor PPMVs’ activities: the Pharmacists Council of Nigeria (PCN); the National Agency for Food and Drug Administration and Control (NAFDAC); and the National Drug Law Enforcement Agency (NDLEA). The PCN is the only organization with the legal mandate to register and license PPMVs. This function used to be handled by the State Ministry of Health (Directorate of Pharmaceutical Services) but in 2004 the Federal Government placed this task under the purview of the PCN who now manages it directly and monitors that PPMVs only sell drugs that have been approved for sale by PPMVs. NAFDAC ensures that only approved drugs are sold and that all drug stocks meet NAFDAC guidelines to reduce the sale of counterfeit drugs, namely that all drugs sold by PPMVs have valid NAFDAC number, manufacturing date and expiring date. The NDLEA is responsible for prevention of narcotic and psychotropic drug sales and monitors drug shops for such. In addition to the three, some PPMVs reported that officials of the Federal and State Ministries of Health and the Nigerian Police also visited them periodically.

### Role of regulatory agencies in regulating PPMVs

All of the regulators interviewed stated that they periodically visit the PPMVs’ shops to monitor their activities. Both the PCN and NAFDAC reported having routine monitoring systems in place across Oyo and Bayelsa states and a clear list of monitoring criteria, which included checking for registration and license, sale of unauthorized or expired drugs, and suitability of business premises, for instance, distance between two drug outlets, availability of adequate space and drug storage in the shop. As the NDLEA is focused on reducing the circulation of illicit and controlled substances, they only visit PPMV shops when they hear reports of illicit activity. The PCN, NAFDAC, and NDLEA all reported having the ability to sanction PPMVs through written warnings, fines, or shop closures. While the PCN, NAFDAC, and NDLEA focused on drug stocks, the State Ministry of Health and malaria programmes focused more on PPMV malaria testing and treatment knowledge and practices. They reported that they often asked questions about how a PPMV would treat a client with fever. While they did not directly sanction PPMVs, they provided the information gathered to non-governmental organizations (NGOs) who worked with PPMVs or PCN for follow-up.

### Frequency of monitoring by regulatory agencies

During the PPMV survey, PPMVs were asked which regulatory agencies, including their local PPMV association, had visited their shops in the past 6 months. Overall, 132 (82.5%) of PPMVs reported being monitored, with the percentage of PPMVs receiving at least one visit varying widely by LGA. Over 95.0% of the shops in both Oyo LGAs reported having at least one monitoring visit in the last 6 months. In Bayelsa LGAs, 32 (80.0%) of the Brass shops and 23 (57.5%) of the Ogbia shops had received a monitoring visit. Table 3 shows LGA PPMV associations reached the greatest proportion of PPMVs with 110 out of 160 (68.8%) of PPMV shops visited in the past 6 months. NAFDAC was the government agency that monitored PPMVs most often covering 36.3% of the PPMVs sampled. The state PPMV association and the State Ministry of Health (SMOH) were all reported to have monitored less than 20% of the PPMVs, The Federal Ministry of Health (FMOH) and the police were other government agencies who monitored.

**Table 3:** Monitoring of PPMVs in the previous six months by regulatory agencies

	Oyo			Bayelsa			Total
	Ibarapa East N (%)	Kajola N (%)	Total	Brass N (%)	Ogbia N (%)	Total	
At least one monitoring visit	38 (95.0)	39 (97.5)	77 (96.3)	32 (80.0)	23 (57.5)	55 (68.8)	132 (82.5)
**By agency**							
LGA PPMV association	30 (75.0)	38 (95.0)	68 (85.5)	25 (62.5)	17 (42.5)	42 (52.5)	110 (68.8)
NAFDAC	16 (40.0)	14 (35.0)	30 (37.5)	14 (35.0)	14 (35.0)	28 (35.0)	58 (36.3)
PCN	30 (75.0)	11 (27.5)	41 (51.3)	1 (2.5)	5 (12.5)	6 (7.5)	47 (29.4)
NDLEA	16 (40.0)	18 (45.0)	34 (42.5)	9 (22.5)	5 (12.5)	14 (17.5)	48 (30.0)
State PPMV association	10(25.0)	13(32.5)	23 (28.8)	2 (5.0)	2 (5.0)	4 (5.0)	27 (16.9)
State Ministry of Health	14(35.0)	12 (30.0)	26 (32.5)	7 (17.5)	0 (0.0)	7 (8.8)	33 (20.6)
**Others**	13 (32.5)	14 (35.0)	27 (33.8)	1 (2.5)	2 (5.0)	3 (3.8)	30 (37.5)

### Capacity building of PPMVs

Most of the regulatory agencies reported providing some form of training for PPMVs. PCN reported providing continuing education to PPMVs every 2 years. NAFDAC and NDLEA both reported conducting seminars related to selling of appropriate drugs. The state governments reported primarily doing training through NGOs that support PPMVs, rather than conducting trainings themselves.

### Successes and challenges in PPMV regulation

Some regulators reported strong working relationships with the PPMV Associations. These organizations mentioned best practices such as having consultative meetings with executives of the PPMV Associations where both organizations could discuss their challenges and come to mutually agreed solutions, and attending PPMV association meetings within their LGAs when possible. Over time the open communication between certain regulatory bodies and the PPMV Associations had resulted in mutual trust and respect. In fact, the associations had even begun to report to regulatory bodies when drug sellers, such as drug hawkers or particular PPMVs, were not adhering to stated guidelines so that the regulatory bodies could investigate.

*“Their associations also have regular meetings what we call consultative meetings with us. They come here to have consultative meetings and they tell us their minds and what is worrying them, the challenges they are facing. It has helped us a lot. For example they come here and say so, so, so, so are not cooperating with us we suspect they might want to be doing the wrong things so please focus on them. That information helps us to go there and monitor them; what is happening you are not going for your meeting, are you doing that? You know it helps us a lot.” – Regulator, Oyo State*

*“At that first time we started, we are like trying to enforce the law and we noticed that along the line they have that fear in us. What we did was to invite the association. We started spelling them our functions, why we are doing this and why we are doing that. I think at the end of the day we were able to get them to understand the reason why sometimes we sanction, punish and sometimes warn them...Overtime, the PPMVs have come to appreciate the regulatory functions of [organization name] which has to do with the fact that if they err they will be sanctioned without exception.”– Regulator, Bayelsa State*

Most of the regulators reported similar challenges in carrying out monitoring of PPMVs. A common challenge reported by regulators was lack of manpower and funding, which hindered organizations’ abilities to routinely monitor PPMVs. For example, one organization in Oyo reported having only two staff members to monitor all PPMVs within the state. Monitoring in Bayelsa state was further complicated by the largely riverine nature of the state; however, by creating desk officers and partnering with the National Youth Service Corps, NAFDAC was able to extend its reach.

*“Here in this particular environment, you’ll all agree with me that apart from Yenagoa, Ogbia and Kolokuma, the rest are riverine areas, so sometimes it’s very difficult for us to cross to those areas. What we’ve done is that we have what we call desk offices in all the local government areas and we even partner with NYSC (National Youth Service Corps) so once in a while we’ve been able to go those places. And with some help from the state government, we’ll be able to monitor effectively as we’re doing here upland.” – Regulator, Bayelsa*

Another common concern was security of regulatory agency staff during monitoring visits. Some organizations had been able to overcome the challenge through building a strong relationship with the PPMV Associations and PPMVs.

*“The first challenge we do encounter is that of security. You know when you regulate somebody’s business, it’s something the person is doing for his life earning and once you touch it he believes you’re just trying to destroy his future or his business. But we’ve been able to overcome that with time because before any place we’re going we go with armed security but now we’ve been able to carry them along and we’ve been able to overcome that challenge” – Regulator, Bayelsa*

Regulators also reported that PPMVs low level of training and their being primarily “business-minded” and seeing themselves only secondarily as health providers led to difficulties in being able to consistently improve their practices.

### Strengthening PPMV associations to support regulation

When asked how PPMV Associations could be strengthened to support the goals of the various regulatory agencies, the regulators primarily responded that they should be provided further training and education so that they could become familiar with key regulations and better understand why these regulations should be followed. One regulator assigned responsibility for training to the local government health department, which she felt should be involved in delivering these trainings.

*“We should continue to educate them you know we educate them on what to do and what not to do. What they should practice and what they should not. Then also by working with them, we will encourage them to have a kind of cooperative that at least will keep their business alive.” – Regulator, Oyo*

Other ways to strengthen PPMV Associations mentioned by the regulators included meeting and collaborating with PPMV Associations and greater recognition of the PPMVs and their associations within the government sector.

*“Government should accord them the recognition they deserve and Government should let them know how important they are in the health sector because they are of importance to the health sector and they are playing a great role; a fact you cannot rule out.” – Regulator, Bayelsa*

*“There is a need for a greater synergy between the Pharmaceutical Society of Nigeria (PSN), Pharmacists Council of Nigeria (PCN) and the PPMVs… After all, if you look around, you will see that the PPMVs are more in the grassroots. So they should be a priority in the health sector.”– Regulator, Bayelsa*

However, one regulator was not supportive of further strengthening PPMV associations to regulate their members, he felt that the PPMV associations were already trying to circumvent government regulation by establishing PPMV Association Monitoring Task Forces.

*“Some of them have taken it upon themselves to set up their own inspectorate unit and boycott the one set up by the government which is illegal. Some of them pay huge amount of money ranging from 75,000 naira to 200,000 naira in some LGA associations, which they call NAPPMED, and they claim that they are coming to register, which they do not do. No association can be bigger than the government, there must be a regulation and you cannot regulate yourself.” – Regulator, Bayelsa*

## Discussion

PPMVs play an important role in the management of common diseases in Nigeria, especially in many rural areas where there are usually few pharmacies and health facilities maybe either non-existent or non-functioning [13, 14]. However, the quality of services at PPMVs’ shops is low, and there is no clear consensus on how to enforce compliance with standards and improve the quality of services at these drug shops [29, 30].

This study revealed the presence of a cohesive association, National Association of Patent and Proprietary Medicine Dealers (NAPPMED), which unites a group of providers that play an important role in the management of common diseases but are yet to be formally integrated into the health care delivery system of Nigeria. Partnerships between NAPPMED and regulatory bodies and other stakeholders could support the rapid scale-up of improved regulation and interventions that could lead to improved quality of care in Nigeria.

A major challenge expressed by the regulatory bodies was inadequate resources to monitor PPMVs as frequently as planned. This is shown in the low frequency of regulatory agency monitoring visits reported by PPMVs. Most regulatory agencies were found to be supportive of PPMV associations monitoring their members and several had already partnered with local chapters to improve regulation, demonstrating that PPMV associations and regulatory agencies can effectively work together. At the national level, key regulatory agencies could work with NAPPMED to establish standard operating procedures and tools to guide PPMV associations in monitoring their members. Summaries of monitoring visits could be shared with the regulatory agencies during joint meetings to aid in determining where additional oversight is required.

In the interviews the PPMV associations reported focusing on enforcing association rules, rather than PCN and other agencies’ regulations. None of the PPMVs in this study were sanctioned by their association for poor quality of care or violating PCN regulations. No member reported being penalized by their association for stocking and selling unapproved drugs and other health commodities, although this has been documented as a widespread practice in other studies [6, 12, 18, 21, 25]. PPMVs also reported that one of their associations’ main functions was to protect them from harassment by regulatory agencies, thus the associations’ increasing regulation of their members may be considered a conflict of interest. Some PPMV associations may fail to conduct monitoring visits or produce inaccurate reports to protect their members. To mitigate this risk, regulatory agencies should continue direct monitoring of a sample of shops and regularly audit the association’s monitoring systems. For example, PCN could regularly check PCN registration and licensing lists against the PPMV association member lists, as well as conducting a spot check of PPMV shops within an LGA. Sieverding et al. also reported the presence of a task office within NAPPMED performing a similar self-regulating function in the three states of Kwara, Kogi and Enugu where their study was conducted [28].

The associations in the LGAs served as a professional support network for members, a finding similar to that of Sieverding and colleagues [28]. The monthly meeting of the associations which was usually well attended by members is an excellent opportunity for capacity building programmes and information sharing to improve the knowledge and competencies of the PPMVs. In our study and many similar others, some association members have formal health training and some are even simultaneously working in formal health facilities [6, 24, 28]. These members tend to be more knowledgeable of health care practices and more compliant with regulatory requirements [6, 24]. Such members could act as peer educators for their colleagues. In line with Nigeria’s task shifting policy [31], regulators and intervention programmes could partner with PPMV associations to rapidly scale up training on common diseases that PPMVs can manage. Such trainings can utilize members of the associations who have formal health training.

One example of interventions where PPMVs could be further capacitated is integrated community case management (iCCM), a programme that targets malaria, diarrhoea, and pneumonia – three diseases that are responsible for about 54% of under-five mortality in Nigeria [32, 33]. PPMVs are increasingly being assessed and engaged to scale up this intervention [24, 34–36]. One intervention that has achieved remarkable success - Enhancing Quality iCCM through Proprietary and Patent Medical Vendors (PPMV) and Partnerships (EQuiPP) - worked actively with NAPPMED in the two study states [34]. The model used in the study can be scaled up. For example, at the national level, iCCM stakeholders and PCN could work with NAPPMED to establish iCCM treatment protocols for use by PPMVs and strengthen association bylaws to include iCCM standards and sanctions for offences. Based on these standards, PPMV training materials could be developed for use during PPMV apprenticeships, orientation courses with PCN, and PPMV association meetings for continuing education. Standard operating procedures and tools could be developed to guide PPMV associations in monitoring iCCM quality of care.

This survey was conducted in 2013 but the findings are still relevant in Nigeria’s current health care context. With increasing loss of confidence in public health facilities due to poor funding, frequent stock out of essential commodities, and recurrent industrial actions by healthcare workers [37, 38] many Nigerians turn to the private sector for their healthcare needs. Though the exact number of PPMVs in Nigeria is unknown, recent studies show they remain the predominant group of private providers of basic healthcare services in the country [3, 6]. Under Nigeria’s task-shifting policies enacted in 2014/2015, PPMVs are increasingly being called upon to act as community-oriented resource persons (CORPs), assisting in treatment for HIV/AIDS, TB, malaria and maternal and child health [31, 33, 39]. .In 2015, the National Malaria Elimination Programme (NMEP) changed their policy to allow PPMVs to carry out RDTs for malaria case management [39]. With this increase in roles and responsibilities, PPMVs need both regulation and support to ensure delivery of high quality care. As this study has demonstrated, local PPMV Associations could be leveraged to rapidly scale-up PPMV oversight, which could lead to improvements in treatment provision.

## Study limitations

The study was conducted in two rural LGAs in two states; NAPPMED in other states and urban locations may operate differently, limiting the countrywide generalizability of our findings. However, there is reason to believe that the constitution and operations of the association may not be significantly different in other places given the national structure of the association. Not all PPMVs in the study LGAs belonged to NAPPMED and it is also possible that other associations apart from NAPPMED exist in other parts of the country and may have a different operating system to influence the type of services they provide in the community. Engaging with such associations may therefore require a different approach other that what we propose for NAPPMED. Evaluation of PPMV associations in more states across the six geopolitical zones in Nigeria could provide more insight into their activities.

## Conclusions

Nigeria needs to rapidly expand coverage of its population with proven health interventions in order to attain target 3.8 of Sustainable Development Goal (SDG) which speaks to achieving universal health coverage through access to quality essential health-care services and access to safe, effective, quality and affordable essential medicines [2]. Quality services at any point of delivery require regular monitoring and oversight by the relevant regulatory authority [40]. PPMVs are crucial in Nigeria’s quest to attain UHC but regulation of their practice is weak and fragmented across multiple organizations. PPMV associations already play a role in “regulating” and monitoring their members and have existing structures that could be used to improve the quality of services they render. Given regulatory agencies’ limited monitoring resources, partnering and strengthening PPMV associations’ monitoring capacity could be a cost-effective method of increasing routine monitoring of PPMV shops to ensure good treatment practices by their members. Research could be done in a few LGAs to determine the feasibility of the proposed PPMV association monitoring system to scale up iCCM as an example and identify key operational challenges. Then further research could be conducted in other locations to determine scalability of the interventions to other programmes that utilize the services PPMVs.

## Supplementary information

**Additional file 1.** PPMV knowledge survey with drug audit. This is the questionnaire used to collect data about the demographics, knowledge of malaria diagnosis and treatment, involvement of PPMV with NAPPMED and to assess the stock of antimalarial medicines in the drug shop.

**Additional file 2.** Focus Group Discussion. This is the guide used for the focus group discussion with the PPMVs about their membership of NAPPMED - benefits and challenges, and knowledge of malaria diagnosis and treatment.

**Additional file 3.** PMV All Staff Interview. This guide was used to interview NAPPMED executives about their positions and functions. It also explored their views about the role their association could play to improve malaria diagnosis and treatment in their locality.

**Additional file 4.** Regulator Key Informant Interview. This is the guide used to interview representatives of regulatory agencies about the specific role of their organization in regulating the activities of PPMVs.

## Data Availability

The datasets generated and analysed during the study are not publicly available as they are owned by the funding organization but may be available from the corresponding author on reasonable request.

## Abbreviations

CORPs: Community-Oriented Resource Persons
iCCM: Integrated Community Case Management
EQuiPP: Enhancing Quality iCCM through Proprietary and Patent Medical Vendors (PPMV) and Partnerships
FGD: Focus group discussion
LGA: Local Government Area
NAFDAC: National Agency for Food and Drug Administration and Control
NMEP: National Malaria Elimination Programme
NDHS: Nigeria Demographic and Health Survey
NDLEA: National Drug Law Enforcement Agency
NAPPMED: National Association of Patent and Proprietary Medicine Dealers
PCN: Pharmacists Council of Nigeria
PPMVs: Patent and Proprietary Medicine Vendors
SDG: Sustainable Development Goal
SMCP: State Malaria Control Programme
SMOH: State Ministry of Health
UHC: Universal Health Coverage
