# Supplementary material for: The potential role of patent and proprietary medicine vendors’ associations in improving the quality of services in Nigeria’s drug shops
Source: BMC Health Serv Res. 2020 Jun 22;20:567. doi: 10.1186/s12913-020-05379-z (PMC7310190; doi:10.1186/s12913-020-05379-z)
Supplement: Supplementary file 1 — Additional file 1. PPMV knowledge survey with drug audit. This is the questionnaire used to collect data about the demographics, knowledge of malaria diagnosis and treatment, involvement of PPMV with NAPPMED and to assess the stock of antimalarial medicines in the drug shop. [file 12913_2020_5379_MOESM1_ESM.pdf]

Informed Consent for PMV Knowledge Survey with Drug Audit:
THIS COPY TO BE GIVEN TO INTERVIEWEE

This project we want you to join is a study. The University of Ibadan (UI) Future Health System Research Group and the Niger Delta University in Nigeria in collaboration with the West African Infectious Disease Institute are carrying out a study titled "Strengthening Patent Medicine Vendors' Associations in Nigeria for Improved Malaria Management (SPANIMM)" which involves collecting information from PMVs through survey on malaria diagnosis and treatment practices and the PMV Association role.

If you choose to participate in this study, we will ask questions about aspects of your work related background and experiences. We will ask questions on how you stock malaria medicines and diagnose and treat malaria and observe some medicines in your shop. We would also like to learn about how you keep records of your activities and seek suggestions on how the PMV Association can improve PMVs malaria diagnosis and treatment practices in this area.

The questions are general but if you find that some questions are not going well with you, please do not feel compelled to answer any of them for any reason. We will talk to you for about 45-60 minutes. You can decide if you want to take part in this survey. Taking part in this study will not cost you or your family anything. You may also leave the survey at any time. You can leave for any reason without any problems. You and your family may not get any direct benefits from being in this study. What you tell us will help us better develop a strategy for improving PMV associations' capacity to influence PMVs malaria knowledge and management practices in line with the revised National Policy on Diagnosis and Treatment of Malaria of 2011. There are no risks involved in your participation in this survey interview. The benefit of this study to you is that you will have an opportunity to talk about your experiences working with the PMV association. As a result of this study, PMVs may be able to diagnose and treat malaria more effectively in rural areas thereby reducing malaria illness and deaths.

We will provide you with a present as a token of our appreciation of your time and effort for participating in this interview.You would not incur any financial costs for being in this study. The only cost of your participation is the time you allow for answering the survey questions. Your name and what you say to us for this study will be kept private as much as the law allows.

Do you have any questions about the study?

If you have any questions about your rights in the study or in case of emergency, you may contact the following persons during the study and in the future:

- Professor Oladimeji Oladepo at the Dept. of Health Promotion & Education, Faculty of Public Health, College of Medicine, University of Ibadan, Ibadan, Oyo State, Nigeria, Phone:0803-326-3302
- Dr. Abisoye Oyeyemi, Department of Community Medicine, Faculty of Clinical Sciences, College of Health Sciences, Niger Delta University Wilberforce Island, Bayelsa State in Nigeria, Phone: 0803-704-9837
- Ms. Sarah Burnett at Accordia Global Health Foundation,1101 14th Street NW, Suite 801, Washington, DC 20005, phone: + 1 202 534 1200

If you agree to participate in this survey interview you can tell us that you agree by repeating these words and then putting your name and signature in the space below.

"I have read the foregoing information, or it has been read to me. I have had the opportunity to ask questions about it and any questions I have asked have been answered to my satisfaction. I voluntarily consent to participate as a subject in this study and understand that I have the right to withdraw from the study at any time without in any way affecting my work position."

Individual PMV Participant's name/ signature	Date	Participant preferred verbal consent confirmed by RA	Date

Informed Consent for
PMV Knowledge Survey with Drug Audit:
THIS COPY TO BE FILED FOR THE PROJECT

This project we want you to join is a study. The University of Ibadan (UI) Future Health System Research Group and the Niger Delta University in Nigeria in collaboration with the West African Infectious Disease Institute are carrying out a study titled "Strengthening Patent Medicine Vendors' Associations in Nigeria for Improved Malaria Management (SPANIMM)" which involves collecting information from PMVs through survey on malaria diagnosis and treatment practices and the PMV Association role.

If you choose to participate in this study, we will ask questions about aspects of your work related background and experiences. We will ask questions on how you stock malaria medicines and diagnose and treat malaria and observe some medicines in your shop. We would also like to learn about how you keep records of your activities and seek suggestions on how the PMV Association can improve PMVs malaria diagnosis and treatment practices in this area.

The questions are general but if you find that some questions are not going well with you, please do not feel compelled to answer any of them for any reason. We will talk to you for about 45-60 minutes. You can decide if you want to take part in this survey. Taking part in this study will not cost you or your family anything. You may also leave the survey at any time. You can leave for any reason without any problems. You and your family may not get any direct benefits from being in this study. What you tell us will help us better develop a strategy for improving PMV associations' capacity to influence PMVs malaria knowledge and management practices in line with the revised National Policy on Diagnosis and Treatment of Malaria of 2011. There are no risks involved in your participation in this survey interview. The benefit of this study to you is that you will have an opportunity to talk about your experiences working with the PMV association. As a result of this study, PMVs may be able to diagnose and treat malaria more effectively in rural areas thereby reducing malaria illness and deaths.

We will provide you with a present as a token of our appreciation of your time and effort for participating in this interview. You would not incur any financial costs for being in this study. The only cost of your participation is the time you allow for answering the survey questions. Your name and what you say to us for this study will be kept private as much as the law allows.

Do you have any questions about the study?

If you have any questions about your rights in the study or in case of emergency, you may contact the following persons during the study and in the future:

- Professor Oladimeji Oladepo at the Dept. of Health Promotion & Education, Faculty of Public Health, College of Medicine, University of Ibadan, Ibadan, Oyo State, Nigeria, Phone:0803-326-3302
- Dr. Abisoye Oyeyemi, Department of Community Medicine, Faculty of Clinical Sciences, College of Health Sciences, Niger Delta University Wilberforce Island, Bayelsa State in Nigeria, Phone: 0803-704-9837
- Ms. Sarah Burnett at Accordia Global Health Foundation,1101 14th Street NW, Suite 801, Washington, DC 20005, phone: + 1 202 534 1200

If you agree to participate in this survey interview you can tell us that you agree by repeating these words and then putting your name and signature in the space below.

"I have read the foregoing information, or it has been read to me. I have had the opportunity to ask questions about it and any questions I have asked have been answered to my satisfaction. I voluntarily consent to participate as a subject in this study and understand that I have the right to withdraw from the study at any time without in any way affecting my work position."

______________________	__________	______________________	__________
Individual PMV Participant's name/ signature	Date	Participant preferred verbal consent confirmed by RA	Date

SERIAL NO: _______________

Tool 2.

PMV Knowledge Survey with Drug Audit:

Introductions

My name is _________________________________. I work for a research group based in the Niger Delta University. We are doing a project funded by the West African Infectious Disease Institute. We have invited you for an interview on issues relating to the PMV Association in this State. This interview is important because it will help us understand how PMV Association operates in this state. We shall be grateful if you are honest in answering all the questions.

**State:**	**LGA:**	**Serial No:**
**Interview Visits**		
**1**	**2**	**3**
**Date**	**Date**	**Date**
**Interviewer**	**Interviewer**	**Interviewer**
**Start Time**	**Start Time**	**Start Time**
**Stop Time**	**Stop Time**	**Stop Time**
**Completed? Yes/No**	**Completed? Yes/No**	**Completed? Yes/No**
**Next Visit Date**	**Next Visit Date**	**Next Visit Date**
**Next Visit Time**	**Next Visit Time**	**Next Visit Time**

QUALIFYING QUESTION:

Have you ever registered with the LGA PMV Association?

IF YES, CONTINUE INTERVIEW

IF NO, VERIFY PMV NAME AND SHOP NAME AGAINST PARTICIPANT LIST.

IF PMV AND SHOP NAME MATCH - CONTINUE INTERVIEW.

IF PMV AND SHOP NAME **DO NOT** MATCH - END INTERVIEW.

SERIAL NO: ______________

Q NO	ITEMS	RESPONSES (MAKE SURE YOU FILL ALL COLUMNS)
**SECTION 1: DEMOGRAPHICS**		
001	**RECORD RESPONDENT'S SEX**	MALE ....1<br>FEMALE 2
002	What was your age as of your last birthday?	☐☐ YEARS
003	What is your ethnic group?	YORUBA.................... ....1<br>IGBO......................... ....2<br>HAUSA ....3<br>URHOBO ....4<br>NEMBE ....5<br>OGBIA ....6<br>OTHER ________________ ....7<br>(SPECIFY)
004	What is your religion?	CHRISTIANITY........... ....1<br>ISLAM ....2<br>TRADITIONAL ....3<br>OTHER ________________ ....4<br>(SPECIFY)
005	What is your highest level of education completed?<br><br>**IF ANY ANSWER EXCEPT TERTIARY GO TO Q 007**	NO FORMAL EDUCATION .... 1<br>RELIGIOUS EDUCATION................. 2<br>ADULT EDUCATION......................... 3<br>PRIMARY .... 4<br>JUNIOR SECONDARY .... 5<br>SENIOR SECONDARY .... 6<br>POST-SECONDARY (GRADE 2)...... 7<br>TERTIARY.. 8 **GO TO Q 006**<br>OTHER ________________ .... 9<br>(SPECIFY)

CONTINUE ON NEXT PAGE

SERIAL NO: ______________

Q NO	ITEMS	RESPONSES<br>(MAKE SURE YOU FILL ALL COLUMNS)
006	IF TERTIARY,<br><br>What type of tertiary education?	ND (OND) .... 1<br>NCE .... 2<br>VOCATIONAL/TECHNICAL .... 3<br>UNIVERSITY FIRST DEGREE<br>(BACHELORS/HND) .... 4<br>UNIVERSITY POSTGRADUATE ...... 5<br>OTHER ________________ .... 6<br>(SPECIFY)
007	Have you ever participated in a PMV apprenticeship program?	YES.. 1<br>NO...2 **GO TO Q013**
008	IF YES,<br><br>How many years did you spend in an apprenticeship?	☐☐ YEARS<br><br>ENTER “0” if less than 1 year
009	Did you complete the apprenticeship?	YES... 1<br>NO... 2 **GO TO Q013**<br>CURRENTLY IN APPRENTICESHIP... 3 **GO TO Q013**
010	What was the year of completion?	☐☐☐☐<br><br>DON’T KNOW/DON’T REMEMBER…..88
011	If yes, were you issued a certificate at the end of the apprenticeship training?	YES... 1<br>NO... 2 **GO TO Q013**
012	**[EVIDENCE OF APPRENTICESHIP CERTIFICATE SEEN]?**	YES... 1<br>NO... 2
013	Have you ever undergone any formal health professional education in a government training school?	YES... 1<br>NO... 2 **GO TO Q015**

CONTINUE TO NEXT PAGE

SERIAL NO: _____________

Q NO	ITEMS	RESPONSES (MAKE SURE YOU FILL ALL COLUMNS)
014	IF YES, what is the type of health professional education, the duration, status, year of completion and evidence?	

		a) Type of education		b) Duration of Training? (years)	c) Is education completed?			d) Year of completion	(e) Evidence of Certification	
Line No.	Education Program (No. of years of training)	Yes	No		Yes	No	Still on	DK=Don't Know	Yes	No
1	Community Health Officer (2)	1	2		1	2	3		1	2
2	Pharmacy Technician (2)	1	2		1	2	3		1	2
3	Auxiliary Nurse (2)	1	2		1	2	3		1	2
4	Junior Community Health Extension Worker (2)	1	2		1	2	3		1	2
5	Senior Community Health Extension Worker (2)	1	2		1	2	3		1	2
6	General Nursing (3)	1	2		1	2	3		1	2
7	Nurse-Midwife (4)	1	2		1	2	3		1	2
8	Environmental Health Officer (2)	1	2		1	2	3		1	2
9	Other, specify: _____________	1	2		1	2	3		1	2

Q NO	ITEMS	RESPONSES
015	Have you ever been trained as a role model mother or a voluntary health care worker?	ROLE MODEL MOTHER…………………………..1<br>OTHER VOLUNTARY HEALTH CARE WORKER ________________……………….... 2<br>(SPECIFY)<br>NO OTHER HEALTH WORKER TRAINING…….3<br>**IF NO OTHER TRAINING, GO TO Q 018**
016	IF YES, Duration of training?<br>**IF LESS THAN 1 YEAR WRITE 00 IN YEARS**<br>**IF LESS THAN 1 MONTH WRITE 00 IN MONTHS AND YEARS**	☐☐ YEARS<br>☐☐ MONTHS<br>☐☐ WEEKS<br>DON'T KNOW/DON'T REMEMBER…..88

SERIAL NO: ______________

Q NO	ITEMS	RESPONSES (MAKE SURE YOU FILL ALL COLUMNS)
017	IF YES, Topics of training?<br><br>**READ ALL RESPONSES**<br><br>**CIRCLE ALL THAT APPLY**	MALARIA .... 1<br>DIARRHEA.. 2<br>ACUTE RESPIRATORY INFECTION... 3<br>OTHER 1________________ .... 4<br>(SPECIFY)<br>OTHER 2________________ .... 5<br>(SPECIFY)
018	How many PMV shops do you have?	☐☐
019	FOR EACH SHOP UP TO 4 SHOPS,<br>**Excluding yourself**, how many other people work in each shop (including regular paid employees, apprentices, and non-paid family members/volunteers)?<br><br>How many apprentices?	(see table below)
020	Is PMV work your primary occupation/employment?	YES ....1**GO TO Q022**<br>NO..2
021	IF NO,<br>What is your primary occupation/employment?	

	Total	# of Apprentices
Shop 1		
Shop 2		
Shop 3		
Shop 4		

Section 2: PMV Profession & Registration

Q NO	ITEMS	RESPONSES
022	How many years have you been selling medicine as PMV?	☐☐ YEARS
023	Are you currently registered with the Patent Medicine Vendors' Association in your LGA?	YES.. 1<br>NO ....2 **GO TO Q026**
024	IF YES,<br>How many years have you been registered?	☐☐ YEARS
025	IF YES,<br>**[EVIDENCE OF THE PMV ASSOC. REGISTRATION AVAILABLE]?**	YES.. 1<br>NO 2

SERIAL NO: ______________

Q NO	ITEMS	RESPONSES (MAKE SURE YOU FILL ALL COLUMNS)
026	Did you pay the full amount of your annual dues to the PMV Association in 2012?	YES............1 **GO TO Q 028**<br>NO .... 2<br>DON'T REMEMBER............ 3
027	IF NO, Why?	
028	How often do you attend PMV Association meetings?	WEEKLY............1<br>FORTNIGHTLY ....2<br>MONTHLY ....3<br>QUARTERLY............4<br>EVERY SIX MONTHS ....5<br>ONCE A YEAR ....6<br>DO NOT ATTEND MEETINGS ....7<br>OTHER ________________ ....8<br>(SPECIFY)
029	How active would you say you are in the PMV Association?<br><br>**By "active" we mean going to meetings and participating in Association activities**	VERY ACTIVE ....1<br>ACTIVE............2<br>SOMEWHAT ACTIVE ....3<br>NOT ACTIVE 4
030	In what ways has the PMV Association assisted you in the past six months ?<br><br>**By "assistance" we mean any financial, technical, material support or appreciation and moral support**<br><br>**CHECK ALL THAT APPLY**	NO ASSISTANCE RECEIVED ....1<br>CASH GIFT............2<br>CASH LOAN............3<br>OTHER PAYMENTS............4<br>EQUIPMENT/ SUPPLY DONATIONS ....5<br>APPRECIATION ....6<br>OTHER ________________ ....7<br>(SPECIFY)
031	Have you ever been sanctioned by the PMV association?	YES............ 1<br>NO ....2 **GO TO Q034**
032	IF YES,<br>How many times?	☐☐<br><br>DON'T KNOW/DON'T REMEMBER…..88

SERIAL NO: _______________

Q NO	ITEMS	RESPONSES (MAKE SURE YOU FILL ALL COLUMNS)	
033	IF YES,<br>What was the offense committed and what was the associated sanction?		
Line no	Offense	Sanction	
1			
2			
3			
4			
034	In what ways has the community assisted you in the past six months?<br><br>**CHECK ALL THAT APPLY**	NO ASSISTANCE RECEIVED............................1<br>REFERRED PATIENTS.....................................2<br>MOBILISED COMMUNITY MEMBERS TO USE SERVICES..3<br>APPRECIATION / RECOGNITION ....4<br>ALERTED PMV TO REGULATORY BODIES....5<br>OTHER _________________ ....6<br>(SPECIFY)	
035	Are you currently registered with the Pharmacists' Council of Nigeria?	YES.. 1<br>NO ....2 **GO TO Q040**	
036	IF YES,<br>How many years have you been registered?	☐☐ YEARS	
037	IF YES,<br>Have you been given a current license to practice from the PCN?	YES...1 **GO TO Q 039**<br>NO 2	
038	IF NO,<br>Why do you not have a current license?		**SKIP TO Q 042**
039	IF YES,<br>**[EVIDENCE OF THE CURRENT PCN LICENSE AVAILABLE]?**	YES…………………………………….1<br>NO……………………………………..2	**SKIP TO Q 042**

SERIAL NO: ______________

Q NO	ITEMS	RESPONSES (MAKE SURE YOU FILL ALL COLUMNS)
040	IF NO,<br>Why are you not registered?	NO BENEFITS....1<br>NOT INTERESTED ....2<br>TOO MUCH SUPERVISION ....3<br>NOT QUALIFIED ....4<br>OTHER ______________ ....5<br>(SPECIFY)
041	If you were given the opportunity to register with the Pharmacists' Council of Nigeria, would you do so?	YES....1<br>NO ....2<br>DON'T KNOW....3
**SECTION 3: PREVIOUS TRAINING ON MALARIA**		
042	Have you received any training on malaria in the last three years?	YES.... 1<br>NO ....2 **GO TO Q 046**<br>DON'T KNOW OR REMEMBER ....88 **GO TO Q 046**
043	IF YES, when was the most recent training on malaria conducted?	☐☐ / ☐☐<br>MONTH YEAR<br>DON'T KNOW OR REMEMBER 88
044	THINKING OF THIS MOST RECENT TRAINING,<br>Who organized the training?	PHARMACISTS' COUNCIL OF NIGERIA (PCN)....1<br>PHARMACEUTICAL SOCIETY OF NIGERIA (PSN) ....2<br>PRIVATE DOCTORS/NURSES ....3<br>SOCIETY FOR FAMILY HEALTH....4<br>MINISTRY OF HEALTH ....5<br>EDUCATIONAL INSTITUTIONS (SCHOOL OF HYGIENE/ HEALTH TECHNOLOGY, ETC.) ....6<br>PMV ASSOCIATION ....7<br>OTHER ______________ ....8<br>(SPECIFY)<br>DON'T KNOW / DON'T REMEMBER....99

CONTINUE ON NEXT PAGE

SERIAL NO: ______________

Q NO	ITEMS	RESPONSES (MAKE SURE YOU FILL ALL COLUMNS)
045	THINKING OF THIS MOST RECENT TRAINING,<br>What topics did the training cover?<br><br>**CIRCLE ALL THAT APPLY**<br>**CAN READ LIST**	SIGN AND SYMPTOMS OF MALARIA......................1<br>MALARIA DIAGNOSIS...2<br>APPROPRIATE DRUGS FOR MALARIA TREATMENT...3<br>WHEN TO REFER PATIENTS..................................4<br>PROMOTION OF INSECTICIDE TREATED BED NETS...5<br>POLICY REGARDING THE ROLE OF PMV IN RAPID DIAGNOSTIC TESTING (RDT).................6<br>POLICY REGARDING THE ROLE OF PMV IN MALARIA TREATMENT ....7<br>OTHER 1 _________________ ....8<br>(SPECIFY)<br>OTHER 2 _________________ ....9<br>(SPECIFY)<br>DON'T KNOW / DON'T REMEMBER.......................99

046	Which of these bodies has monitored your shop in the last six months, if any?<br>Any others?	Monitors members?		Who monitors most often?	What does each body monitor?<br><br>1=Check for expired drugs<br>2= Check for banned drugs<br>3=Check for fake drugs/NAFDAC number<br>4=Check for shop cleanliness<br>5=Check for registration status<br>6=Check for license<br>7=Others, specify____________
Line No.	Monitoring Bodies	Yes	No	**TICK ONE**	**Include #s for all that apply**
1	Federal Ministry of Health (FMOH)	1	2		
2	Pharmacists' Council of Nigeria (PCN)	1	2		
3	LGA PMV Association	1	2		
4	State PMV Association	1	2		
5	State Ministry of Health	1	2		
6	Pharmaceutical Society of Nigeria (PSN)	1	2		
7	NDLEA	1	2		
8	NAFDAC	1	2		
9	Police	1	2		
10	Others, Specify: ________________________	1	2		
11	Others, Specify: ________________________	1	2		

SERIAL NO: ______________

Q NO	ITEMS	RESPONSES (MAKE SURE YOU FILL ALL COLUMNS)
**SECTION 4:**	**KNOWLEDGE OF MALARIA DIAGNOSIS AND TREATMENT**	
047	A child of 24 months old is brought to your shop by her mother. The mother told you that the child has had a fever in the last two days.<br><br>What is the best way to diagnose whether the child is suffering from malaria?<br><br>***Please give only ONE answer***<br><br>**DO NOT READ RESPONSES**	TAKE BLOOD SAMPLE/USE RAPID DIAGNOSTIC TEST .... 1<br>FEEL TEMPERATURE WITH HAND .... 2<br>TAKE TEMPERATURE WITH THERMOMETER .... 3<br>REFER TO HEALTH FACILITY .... 4<br>NO ACTION TO DIAGNOSE .... 5<br>OTHER _________________ (SPECIFY) .... 6<br>DON'T KNOW 99
048	If you determined that the child had malaria, what would you give to the mother to treat her child?<br><br>**WRITE IN UP TO 4 TREATMENTS.**<br>**DO NOT READ DOSAGE CATEGORIES**	

Line No.	TREATMENTS	What is the recommended dosage for a 2-year old child?		
		No. of Tabs/ Spoonfuls	No. of Times per Day	No. of Days
1				
2				
3				
4				

SERIAL NO: ______________

Q NO	ITEMS	RESPONSES (MAKE SURE YOU FILL ALL COLUMNS)
049	Please list the danger signs that a child under 2 years old will show that would suggest he/she is having severe malaria.<br><br>Any others?<br><br>**DO NOT READ RESPONSES**<br><br>**CHECK ALL THAT APPLY**	CONVULSIONS .... 1<br>COMA .... 2<br>SEVERE VOMITING .... 3<br>INABILITY TO EAT, DRINK OR BREASTFEED .... 4<br>INABILITY TO SIT OR STAND .... 5<br>DIFFICULTY BREATHING/FAST BREATHING .... 6<br>PERSISTENT FEVER .... 7<br>LACK OF IMPROVEMENT OR WORSENING OF SYMPTOMS AFTER TWO DAYS .... 8<br>OTHER1________________ .... 9<br>(SPECIFY)<br>OTHER2________________ .... 10<br>(SPECIFY)<br>OTHER3________________ .... 11<br>(SPECIFY)<br>DON'T KNOW ANY DANGER SIGNS 99
50	If a child visited your shop with signs of severe malaria what would you do?<br><br>Anything else?<br><br>**DO NOT READ RESPONSES**<br><br>**CHECK ALL THAT APPLY**	PLACE TEPID SPONGE ON CHILD .... 1<br>REFER TO HEALTH FACILITY .... 2<br>GIVE PARACETAMOL .... 3<br>OTHER1________________ .... 4<br>(SPECIFY)<br>OTHER2________________ .... 5<br>(SPECIFY)<br>DON'T KNOW 99
051	A man aged 32 years comes into your shop, complaining that he has been unwell with fever for 4 days now. The fever shows no sign of getting better.<br><br>What is the best way to diagnose whether the man is suffering from malaria?<br><br>***<u>Please give only ONE answer</u>***<br><br>**DO NOT READ RESPONSES**	TAKE BLOOD SAMPLE/USE RAPID DIAGNOSTIC TEST .... 1<br>FEEL TEMPERATURE WITH HAND .... 2<br>TAKE TEMPERATURE WITH THERMOMETER .... 3<br>REFER TO HEALTH FACILITY .... 3<br>NO ACTION TO DIAGNOSE .... 3<br>OTHER1________________ .... 4<br>(SPECIFY)<br>OTHER2________________ .... 5<br>(SPECIFY)<br>DON'T KNOW 99

SERIAL NO: ______________

Q NO	ITEMS	RESPONSES (MAKE SURE YOU FILL ALL COLUMNS)		
052	If you determined that a 32 year old man had malaria, what would you give him to treat malaria?<br>**WRITE IN UP TO 4 TREATMENTS.**<br>**DO NOT READ DOSAGE CATEGORIES**			
Line No.	TREATMENTS	What is the recommended dosage for a 32 year old man?		
		No. of Tabs	No. of Times per Day	No. of Days
1				
2				
3				
4				
053	A 23 year old pregnant woman comes into your shop, complaining that she has had fever and headache for two days. She is 4 months pregnant.<br><br>What is the best way to diagnose whether the woman is suffering from malaria?<br><br>***<u>Please give only ONE answer</u>***<br><br>**DO NOT READ RESPONSES**	TAKE BLOOD SAMPLE/USE RAPID DIAGNOSTIC TEST ....1<br>FEEL TEMPERATURE WITH HAND ....2<br>TAKE TEMPERATURE WITH THERMOMETER..3<br>REFER TO HEALTH FACILITY................................3<br>NO ACTION TO DIAGNOSE....................................3<br>OTHER1_________________ ....4<br>(SPECIFY)<br>OTHER2_________________ ....5<br>(SPECIFY)<br>DON'T KNOW..99			
054	If you determined that a 23 year old pregnant woman had malaria, what you would you give her to treat malaria?<br>**WRITE IN UP TO 4 TREATMENTS.**<br>**DO NOT READ DOSAGE CATEGORIES**			
Line No.	TREATMENTS	What is the recommended dosage for a 23 year old pregnant woman?		
		No. of Tabs	No. of Times per Day	No. of Days
1				
2				
3				
4				

SERIAL NO: ______________

Q NO	ITEMS	RESPONSES (MAKE SURE YOU FILL ALL COLUMNS)		
055	What would you give to a pregnant woman to **PREVENT** malaria, if anything?<br>**WRITE IN UP TO 4 TREATMENTS. DO NOT READ DOSAGE CATEGORIES**<br>**WRITE "NO TREATMENT" IF PMV WOULD GIVE NO TREATMENT.**			
Line No.	TREATMENTS	What is the recommended dosage for a pregnant woman?		
		No. of Tabs	No. of Times per Day	No. of Days
1				
2				
3				
4				

Q NO	ITEMS	Does the Government recommend this treatment for curing uncomplicated malaria?			Is it effective at curing uncomplicated malaria?		
056	I am going to read a list of treatments.<br><br>Please tell me which ones:<br>a) the government recommends for curing uncomplicated malaria and<br>b) which ones you believe are effective at curing uncomplicated malaria.						
Line No.	Treatments	Yes	No	Don't Know	Yes	No	Don't Know
1	Arthemeter-Lumefantriine	1	2	3	1	2	3
2	Chloroquine	1	2	3	1	2	3
3	Sulphadoxine-pyrimethamine (SP)	1	2	3	1	2	3
4	Herbal Fever Mixture	1	2	3	1	2	3
5	Artesunate-Amodiaquine	1	2	3	1	2	3
6	ArtesunateMonotherapy	1	2	3	1	2	3
7	Paracetamol	1	2	3	1	2	3
8	Quinine	1	2	3	1	2	3

SERIAL NO: ______________

Q NO	ITEMS	RESPONSES<br>(MAKE SURE YOU FILL ALL COLUMNS)
057	Are there any other drugs that the government recommends for curing malaria?	YES .... 1<br>NO ....2**GO TO Q 059**<br>DON'T KNOW.................................3**GO TO Q 059**
058	IF YES,<br>What other drugs does the government recommend for curing malaria?	1.________________________________<br>2.________________________________<br>3.________________________________
059	Are there any other drugs that you know of that are effective at curing malaria?	YES .... 1<br>NO ....2**GO TO Q 061**<br>DON'T KNOW.................................3**GO TO Q 061**
060	IF YES,<br>What other drugs do you know of that are effective at curing malaria?	1.________________________________<br>2.________________________________<br>3.________________________________
061	SHOW THE PMV THE CHILD BRAND (UNDER 5 YEARS) OF ACT "ARTHEMETER –LUMENFANTRINE"<br><br>What is the recommended dosage for a 2-year old child?<br><br>**DO NOT READ DOSAGE CATEGORIES**<br><br>**DO NOT RESTRICT PMV FROM LOOKING UP DOSAGE INFORMATION**	☐☐ TABS<br>☐☐ # TIMES PER DAY<br>☐☐ DAYS<br><br>OTHER _________________......................888<br>(SPECIFY)<br>DON'T KNOW.......................................999
062	SHOW THE PMV THE CHILD BRAND (UNDER 5 YEARS) OF ACT"ARTESUNATE-AMODIAQUINE"<br><br>What is the recommended dosage for a 2-year old child?<br><br>**DO NOT READ DOSAGE CATEGORIES**<br><br>**DO NOT RESTRICT PMV FROM LOOKING UP DOSAGE INFORMATION**	☐☐ TABS<br>☐☐ # TIMES PER DAY<br>☐☐ DAYS<br><br>OTHER _________________......................888<br>(SPECIFY)<br>DON'T KNOW.......................................999

SERIAL NO: _______________

Q NO	ITEMS	RESPONSES (MAKE SURE YOU FILL ALL COLUMNS)
063	SHOW THE PMV THE ADULT BRAND (14+ YEARS) OF ACT "ARTHEMETER – LUMENFANTRINE"<br><br>What is the recommended dosage for an adult?<br><br>**DO NOT READ DOSAGE CATEGORIES**<br><br>**DO NOT RESTRICT PMV FROM LOOKING UP DOSAGE INFORMATION**	☐☐ TABS<br>☐☐ # TIMES PER DAY<br>☐☐ DAYS<br><br>OTHER ________________...................888<br>(SPECIFY)<br>DON'T KNOW.......................................999
064	SHOW THE PMV THE ADULT BRAND (14+ YEARS) OF ACT"ARTESUNATE-AMODIAQUINE"<br><br>What is the recommended dosage for an adult?<br><br>**DO NOT READ DOSAGE CATEGORIES**<br><br>**DO NOT RESTRICT PMV FROM LOOKING UP DOSAGE INFORMATION**	☐☐ TABS<br>☐☐ # TIMES PER DAY<br>☐☐ DAYS<br><br>OTHER ________________...................888<br>(SPECIFY)<br>DON'T KNOW.......................................999
**SECTION 5: MALARIA MEDICINE STOCKS**		
065	Does the shop have an "inner room"?<br>(i.e. consulting room where PMV sees clients)	YES .... 1<br>NO .... 2**GO TO Q 067**
066	IF YES,<br>**[EVIDENCE OF CONSULTING ROOM]?**	YES .... 1<br>NO 2
067	Do you have at least one Insecticide Treated Net or Long Lasting Insecticide Treated Net (ITN/LLIN) in stock?	YES .... 1<br>NO .... 2**GO TO Q069**<br>DON'T KNOW............................ 99
068	IF YES,<br>How many?<br><br>**COUNT THE NUMBER OF NETS**	TOTAL COUNT: ____________<br><br>CAN'T FIND ANY...................88
069	Do you have at least one malaria rapid diagnostic test kit in your shop?<br><br>**IF THEY ARE UNSURE, SHOW AN EXAMPLE TEST KIT**	YES .... 1<br>NO .... 2**GO TO Q 071**<br>DON'T KNOW............................ 99

SERIAL NO: _______________

Q NO	ITEMS	RESPONSES (MAKE SURE YOU FILL ALL COLUMNS)
070	IF YES,<br>How many?<br><br>**COUNT THE NUMBER OF RDTs**	TOTAL COUNT: ____________<br><br>CAN'T FIND ANY………………..88
071	**[EVIDENCE OF PREPACKAGED ARTEMISININ COMBINATION THERAPY (ACT) PACKS IN SHOP?]**<br><br>**CHECK LIST OF ACTs TO CONFIRM**	YES .... 1<br>NO .... 2**GO TO Q 074**<br>DON'T KNOW............................. 99

072	**ACT DRUG STOCKS**<br>Now I'd like to ask you a few questions about the ACT drugs you have in stock.<br>• What ACT drugs do you have in stock?<br>• How many packs do you have for adults and children?<br>• What is the price per pack?<br>• How many packs have you sold in the last seven days?

Line No	**ACT Drug Names** **CHECK LIST OF ACTs TO CONFIRM**		a) No. of ACT packsin stock SYRUP = ONE PACK		b) Price per pack		c) Number of packs sold in the last 7 days			
	**Brand Name**	**Formulation?** (i.e. AA/AL/ OTHER)	Adult (14+)	Child (Under 5)	Adult (14+)	Child (Under 5)	Adult (14+)	Child (Under 5)	RECORDED? Yes	No
1									1	2
2									1	2
3									1	2
4									1	2
5									1	2
6									1	2

073	What is the total number of prepackaged ACTsthat you have sold in the last 3 monthsand for children (Under 5), for adults (14+) and overall?<br><br>**WRITE "DK' FOR DON'T KNOW**	TOTAL NO. SOLD IN 3 MONTHS	#	RECORDED? Yes	No
		CHILD(Under 5)?		1	2
		ADULT(14+)		1	2
		OVERALL		1	2

SERIAL NO: ______________

Q NO	ITEMS	RESPONSES (MAKE SURE YOU FILL ALL COLUMNS)
**SECTION 6: RECORD KEEPING**		
074	Which types of records do you keep in your shop?	(see record table below)

RECORD	Record Kept?		Evidence?	
	Yes	No	Yes	No
TOTAL SALES	1	2	1	2
ACT SALES	1	2	1	2
PATIENT REFERRAL	1	2	1	2
MEDICINES DISPENSED	1	2	1	2
DIAGNOSES	1	2	1	2
MEDICINE PRICES	1	2	1	2
OTHER, SPECIFY: _______________.	1	2	1	2

Q NO	ITEMS	RESPONSES
**SECTION 7: NATIONAL ANTI-MALARIAL POLICY**		
075	Have you heard about the 2011 National Policy on Diagnosis and Treatment of Malaria?<br><br>**SHOW THEM A COPY OF THE GUIDELINES**	YES.. 1<br>NO ....2**GO TO Q 079**<br>DON'T KNOW/ DON'T REMEMBER 99
076	IF YES,<br>Have you ever seen a copy of the 2011 National Policy on Diagnosis and Treatment of Malaria?	YES.. 1<br>NO ....2**GO TO Q 078**<br>DON'T KNOW/ DON'T REMEMBER 99
077	IF YES,<br>Have you read a copy of the 2011 National Policy on Diagnosis and Treatment of Malaria?	YES.. 1<br>NO .... 2<br>DON'T KNOW/ DON'T REMEMBER 99
078	IF YES,<br>Have you read a pamphlet which describes the 2011 National Policy on Diagnosis and Treatment of Malaria?	YES.. 1<br>NO .... 2<br>DON'T KNOW/ DON'T REMEMBER 99

SERIAL NO: _______________

Q NO	ITEMS	RESPONSES (MAKE SURE YOU FILL ALL COLUMNS)
**SECTION 8: IMPROVING PMV MALARIA DIAGNOSIS & TREATMENT PRACTICES**		
079	Have you ever diagnosed malaria using a rapid diagnostic test?<br><br>**SHOW AN EXAMPLE OF THE RDT TEST KIT**	YES ....1<br>NO....................2<br>DON'T KNOW/ DON'T REMEMBER....................99
080	What are the biggest difficulties you face in your job in respect to using RDTs to **<u>diagnose</u>**fevers/Malaria?<br><br>**DO NOT READ OUT OPTIONS**<br><br>**CHECK ALL THAT APPLY**	LACK OF TRAINING / KNOWLEDGE ....1<br>LACK OF SUPERVISION ....2<br>NO ACCESS TO PURCHASE RDT TEST KITS ......3<br>INADEQUATE NUMBER OF CLIENTS ....4<br>TOO MUCH REGULATION ....5<br>CLIENTS NOT AWARE OF RDTS ....6<br>OTHER _________________....................7<br>(SPECIFY)
081	What are the biggest difficulties you face in your job in respect to **<u>treatment</u>**of fevers/Malaria?<br><br>**DO NOT READ OUT OPTIONS**<br><br>**CHECK ALL THAT APPLY**	LACK OF TRAINING / KNOWLEDGE ....1<br>LACK OF SUPERVISION ....2<br>LACK OF DRUGS ....3<br>INADEQUATE NUMBER OF CLIENTS ....4<br>TOO MUCH REGULATION ....5<br>CLIENTS NOT AWARE OF APPROPRIATE MALARIA TREATMENT....................6<br>OTHER _________________....................7<br>(SPECIFY)
082	Do you have any job aids (e.g. charts/booklet) displayed in your shop which describe the signs and symptoms of malaria OR how to treat this disease?	YES ....1<br>NO.................... 2**GO TO Q 084**
083	IF YES,<br><br>**[EVIDENCE OF JOB AIDS IN SHOP]?**	YES ....1<br>NO....................2
084	What would help to improve a) the use of rapid diagnostic tests for malaria and b) malaria treatment at your shop, if anything?	1. ______________________________<br>2. ______________________________<br>3. ______________________________

SERIAL NO: _______________

Q NO	ITEMS	RESPONSES (MAKE SURE YOU FILL ALL COLUMNS)
085	**Interviewee's Comments**	

We have come to the end of the interview.

Thank you for taking the time to talk with us.

**Interviewer's comments**
