# Supplementary material for: The potential role of patent and proprietary medicine vendors’ associations in improving the quality of services in Nigeria’s drug shops
Source: BMC Health Serv Res. 2020 Jun 22;20:567. doi: 10.1186/s12913-020-05379-z (PMC7310190; doi:10.1186/s12913-020-05379-z)
Supplement: Supplementary file 2 — Additional file 2. Focus Group Discussion. This is the guide used for the focus group discussion with the PPMVs about their membership of NAPPMED - benefits and challenges, and knowledge of malaria diagnosis and treatment. [file 12913_2020_5379_MOESM2_ESM.pdf]

Informed Consent for PMV Focus Group Discussions – Registered & Non-Registered
THIS COPY TO BE GIVEN TO INTERVIEWEE

This project we want you to join is a study. The University of Ibadan (UI) Future Health System Research Group and the Niger Delta University in Nigeria in collaboration with the West African Infectious Disease Institute are carrying out a study titled "Strengthening Patent Medicine Vendors' Associations in Nigeria for Improved Malaria Management (SPANIMM)" which involves conducting Focus Group discussions with Patent Medicine vendors on issues relating to the business of patent medicine selling and malaria treatment, and the role of the PMV associations in this area. This discussion is important because it will help us understand the role that patent medicine vendors play in the treatment of malaria, which is a major problem in many parts of Nigeria.

If you choose to contribute to this group discussion, we will ask questions about the PMV Association membership, association's priorities, rules, enforcement, types of support received and association performance. We will ask about achievements and challenges faced by the associations and desired additional services or activities that the association could offer. We want to learn more about the potential of the PMV Associations to improve malaria diagnosis and treatment among PMVs and in what areas PMV Associations need to be strengthened in order to improve malaria diagnosis and treatment. There is no right or wrong answer to any of the questions that will be asked and we do not require your names for this discussion. This discussion session may be taped on a voice recorder if you and other participants agree, so that we do not miss out some of the important things that are said.

The discussion questions are general but if you find that some questions are not going well with you, please do not feel compelled to contribute answers for any of them for any reason. We will discuss with the group participants in which you are a part, for about 45 -60 minutes. You can decide if you want to take part in this discussion. Taking part in this group discussion will not cost you or your family anything. You may also leave the discussion at any time. You can leave for any reason without any problems. You and your family may not get any direct benefits from participating in this discussion. What you tell us will help us to better understand rural LGA PMV Associations' capacity, operations and interest in improving malaria diagnosis and treatment provided by PMV shops in accordance with the Federal Republic of Nigeria 2011 National Policy on Diagnosis and Treatment of Malaria. There are no risks involved in your participation in this focus group discussion. The benefit of this study to you is that you will have an opportunity to contribute your views and opinions on PMV diagnosis and treatment of malaria and how best to position the PMV association to play an enhanced role. As a result of this study, PMVs may be able to diagnose and treat malaria more effectively in rural areas thereby reducing malaria illness and deaths.

We will provide you with a present as a token of our appreciation of your time and effort for participating in the focus group discussion. You would not incur any financial costs for being in this study. The only cost of your participation is the time you allow for answering the discussion questions. Your name and what you say to us for this study will be kept private as much as the law allows.

Do you have any questions about the study?

If you have any questions about your rights in the study or in case of emergency, you may contact the following persons during the study and in the future:

- Professor Oladimeji Oladepo at the Dept, of Health Promotion & Education, Faculty of Public Health, College of Medicine, University of Ibadan, Ibadan, Oyo State, Nigeria, Phone:0803-326-3302
- Dr. Abisoye Oyeyemi, Department of Community Medicine, Faculty of Clinical Sciences, College of Health Sciences, Niger Delta University Wilberforce Island, Bayelsa State in Nigeria, Phone: 0803-704-9837
- Ms. Sarah Burnett at Accordia Global Health Foundation,1101 14th Street NW, Suite 801, Washington, DC 20005, phone: + 1 202 534 1200

If you agree to participate in this discussion you can say that you agree and we will record your response on the tape recorder.

Tool 4a.

Focus Group Discussion Guide for Registered Patient Medicine Vendors (PMVs)

Introductions

My name is _______________ and I am ________________.We work with a research group based in the Niger Delta University. We are doing a project funded by the West African Infectious Disease Institute. We have invited you all to discuss issues relating to the business of patent medicine selling as it relates to malaria treatment in this area. This discussion is important because it will help us understand the role that patent medicine vendors play in treatment of malaria, which is a major problem in many parts of Nigeria. Your answers to all the questions we will ask will be kept secret. There is no right or wrong answer so everyone is encouraged to make his/her contributions. We do not require your names for this discussion. We shall be grateful if you are honest in answering all the questions. If you agree, we would like to record the discussions on a tape recorder to ensure that we do not forget all that we discuss today.

Note takers **must** record the following in notes:

1. Date of Interview
2. Start time of interview
3. Stop time of interview
4. Interviewer Name
5. Note taker Name
6. Interview Tool used
7. Organization of Interviewee
8. Gender of Interviewee

1. Who can become a member of the PMV Association (NAPPMED)? How does someone become a member of the PMV Association (NAPPMED)?

   a) If there is a fee, how much must one pay to become a member of NAPPMED?

2. Why do PMVs join the PMV association?

3. Why are some PMVs not members of the PMV Association? What would it take for them to join the PMV Association?

4. What would cause PMVs to leave their Association?

5. What are the current priorities of your PMV association (NAPPMED)? Are you receiving any assistance from the PMV Association? If yes, please describe. **Probe:**

   a) What kind of support? How often is support received? Can you give a specific example of how this assistance has helped you?

6. What can you say about performance of the PMV association you belong to?

7. What are the association's rules?

    a. If there are drugs that you are not supposed to sell, what are those drugs?

    b. How does the association enforce those rules? How are PMVs who violate the rules punished? Can they give any examples of past enforcements? **Probe, if there are any punishments:**

        i. How effective are the punishments?

        ii. Is there anything that would cause a PMV to lose their membership?

8. What are the achievements of the PMV association to date?

9. What additional services or activities do you wish that your PMV Association could offer, if any and how this should be done?

10. What are the greatest challenges faced by PMVs? How has the PMV Association addressed this issue? What is the outcome? **Probe for:**

    a. In processing government registration
    b. With regulatory bodies, probe for police
    c. With community people who had problems with medicines purchased from PMVs
    d. In procuring quality anti-malarial medicines
    e. With maintaining adequate supply of antimalarials
    f. In procuring ITN

11. Are you aware of the new antimalarial treatment guidelines? What do the anti-malaria treatment guidelines say:

    a. About diagnosing malaria
    b. About treating malaria, including uncomplicated vs. severe malaria
    c. About the role PMVs should play in diagnosing and treating malaria?

If they are not aware of the guidelines, inform them. Show a copy of the guidelines.

12. The government anti-malaria treatment guidelines recommend that all malaria suspects be tested for malaria before being prescribed treatment. New rapid diagnostic tests for malaria exist which allow trained lay workers to conduct this testing.

    a. What are your feelings about this guideline?
    b. What barriers would PMVs have in using these rapid diagnostic tests with patients?
    c. What challenges would PMVs face in encouraging clients to purchase RDTs?
    d. What type of incentives could the PMV associations propose to make PMVs conduct rapid diagnostic tests for all suspected malaria cases before treatment?

13. ACTs are the government recommended first line medicines for treating malaria patients.

    a. What are your feelings about this guideline? ACT vs. older drugs?
    b. What challenges would PMVs face in encouraging clients to choose ACTs over other anti-malarial drugs?
    c. What type of incentives could the PMV associations propose to make PMVs prescribe ACTs as first line treatment for malaria?

14. What are the greatest challenges that PMVs face in diagnosing and treating malaria?

15. The main objective of this project is to build the capacity of PMV associations to improve malaria diagnosis and treatment practices among PMVs through pilot intervention(s).

    a. What are your feelings about this objective?

16. In what ways can the PMV association be strengthened to play a greater role in malaria diagnosis and treatment? **Probe for:**

    a. Competence through continuing professional development
    b. Improved governance
    c. Regulations
    d. Information management
    e. Facilities
    f. Antimalarial medicine procurement and quality
    g. Financing PMV Business
    h. Service delivery

We have come to the end of the discussion and we thank you for your contributions
