# Supplementary material for: The potential role of patent and proprietary medicine vendors’ associations in improving the quality of services in Nigeria’s drug shops
Source: BMC Health Serv Res. 2020 Jun 22;20:567. doi: 10.1186/s12913-020-05379-z (PMC7310190; doi:10.1186/s12913-020-05379-z)
Supplement: Supplementary file 3 — Additional file 3. PMV All Staff Interview. This guide was used to interview NAPPMED executives about their positions and functions. It also explored their views about the role their association could play to improve malaria diagnosis and treatment in their locality. [file 12913_2020_5379_MOESM3_ESM.pdf]

Informed Consent for PMV All Staff Interviews
THIS COPY TO BE GIVEN TO INTERVIEWEE

This project we want you to join is a study. The University of Ibadan (UI) Future Health System Research Group and the Niger Delta University in Nigeria in collaboration with the West African Infectious Disease Institute are carrying out a study titled “Strengthening Patent Medicine Vendors' Associations in Nigeria for Improved Malaria Management (SPANIMM)” which involves collecting information from the principal officers (Chairman, Secretary, Treasurer, Public Relations Officer, Head of Task Force) of the PMV Associations.

If you choose to participate in this study, we will ask you questions about the PMV Association and your tasks and responsibilities. We would like to know about the services you render to members, how you make decisions and the association’s finances. We would like to learn from you about how the members of your association are monitored and how your association works with government regulatory bodies and other associations. We will ask questions on the capacities of your association that could be strengthened. We do not require your names for this interview.

The questions are general but if you find that some questions are not going well with you, please do not feel compelled to answer any of them for any reason. We will talk to you for about 45 -60 minutes. You can decide if you want to take part in this interview. Taking part in this study will not cost you or your family anything. You may also leave the interview at any time. You can leave for any reason without any problems. You and your family may not get any direct benefits from being in this study. What you tell us will help us better understand partnership issues and assist in developing a pilot intervention(s) for PMV associations to enhance PMVs diagnosis and treatment of malaria in line with the revised 2011 National Policy on Diagnosis and Treatment of Malaria. There are no risks involved in your participation in this survey interview.

The benefit of this study to you is that you will have an opportunity to talk about your experiences concerning the PMV association. As a result of this study, PMVs may be able to diagnose and treat malaria more effectively in rural areas thereby reducing malaria illness and deaths.

We will provide you with a present as a token of our appreciation of your time and effort for participating in this interview. You would not incur any financial costs for being in this study. The only cost of your participation is the time you allow for answering the interview questions.

Your name and what you say to us for this study will be kept private as much as the law allows.

Do you have any questions about the study?

If you have ny questions about your rights in the study or in case of emergency, you may contact the following persons during the study and in the future:

- Professor Oladimeji Oladepo at the Dept, of Health Promotion & Education, Faculty of Public Health, College of Medicine, University of Ibadan, Ibadan, Oyo State, Nigeria, Phone:0803-326-3302
- Dr. Abisoye Oyeyemi, Department of Community Medicine, Faculty of Clinical Sciences, College of Health Sciences, Niger Delta University Wilberforce Island, Bayelsa State in Nigeria, Phone: 0803-704-9837
- Ms. Sarah Burnett at Accordia Global Health Foundation,1101 14th Street NW, Suite 801, Washington, DC 20005, phone: + 1 202 534 1200

If you agree to participate in this interview you can say that you agree and we will record your response on the tape recorder.

Tool 6.

Patent Medicine Vendors' Association All Staff Interview

Introduction

My name is ____________________________and my companion is______________________. We work with a research group based in the Niger Delta University. We are doing a project funded by the West African Infectious Diseases Institute. We want to ask you some questions on issues relating to the PMV Association in this local government area. This interview is important to help us understand your role as an executive member of the association and how your position can help to improve the functioning of your association, with emphasis on the treatment of malaria. Your responses to all these questions will be kept secret. We shall be grateful if you are honest in answering all the questions. If you agree, we would like to capture this interview on a tape recorder to ensure that we do not miss any part of the interview. Thank you.

**State:**	**LGA:**	**Serial No:**
**Interview Visits**		
**1**	**2**	**3**
**Date**	**Date**	**Date**
**Interviewer**	**Interviewer**	**Interviewer**
**Start Time**	**Start Time**	**Start Time**
**Stop Time**	**Stop Time**	**Stop Time**
**Completed? Yes/No**	**Completed? Yes/No**	**Completed? Yes/No**
**Next Visit Date**	**Next Visit Date**	**Next Visit Date**
**Next Visit Time**	**Next Visit Time**	**Next Visit Time**

THIS FIRST SECTION TO BE FILLED IN BY THE NOTETAKER

Q NO	ITEMS	RESPONSES (MAKE SURE YOU FILL ALL COLUMNS)
**SECTION 1: DEMOGRAPHICS**		
001	**RECORD RESPONDENT'S SEX**	MALE .... 1<br>FEMALE.. 2
002	What was your age as of your last birthday?	☐☐ YEARS
003	What is your ethnic group?	YORUBA.................... .... 1<br>IGBO .... 2<br>HAUSA....................... .... 3<br>URHOBO .... 4<br>NEMBE .... 5<br>OGBIA....................... .... 6<br>OTHER _________________ .... 7<br>(SPECIFY)
004	What is your religion?	CHRISTIANITY .... 1<br>ISLAM .... 2<br>TRADITIONAL .... 3<br>OTHER _________________ .... 4<br>(SPECIFY)
005	What is your highest level of education completed?<br><br>**IF ANY ANSWER EXCEPT TERTIARY GO TO Q 007**	NO FORMAL EDUCATION ....1<br>RELIGIOUS EDUCATION ....2<br>ADULT EDUCATION ....3<br>PRIMARY..4<br>JUNIOR SECONDARY......................5<br>SENIOR SECONDARY ....6<br>POST-SECONDARY (GRADE 2)......7<br>TERTIARY ....8 **GO TO Q 006**<br>OTHER _________________ ....9<br>(SPECIFY)

CONTINUE ON NEXT PAGE

Q NO	ITEMS	RESPONSES (MAKE SURE YOU FILL ALL COLUMNS)
006	IF TERTIARY,<br><br>What type of tertiary education?	ND (OND)...1<br>NCE..2<br>VOCATIONAL/TECHNICAL ....3<br>UNIVERSITY FIRST DEGREE<br>(BACHELORS/HND)..........................4<br>UNIVERSITY POSTGRADUATE.......5<br>OTHER ________________ ....6<br>(SPECIFY)
007	Have you ever participated in a PMV apprenticeship program?	YES ....1<br>NO .... 2 **GO TO Q013**
008	IF YES,<br><br>How many years did you spend in an apprenticeship?	☐☐ YEARS<br><br>ENTER "0" if less than 1 year
009	Did you complete the apprenticeship?	YES .... 1<br>NO .... 2 **GO TO Q013**<br>CURRENTLY IN APPRENTICESHIP ... 3 **GO TO Q013**
010	What was the year of completion?	☐☐☐☐<br><br>DON'T KNOW/DON'T REMEMBER…..88
011	If yes, were you issued a certificate at the end of the apprenticeship training?	YES .... 1<br>NO .... 2 **GO TO Q013**
012	**[EVIDENCE OF APPRENTICESHIP CERTIFICATE SEEN]?**	YES .... 1<br>NO 2
013	Have you ever undergone any formal health professional education in a government training school?	YES .... 1<br>NO .... 2 **GO TO Q015**

CONTINUE TO NEXT PAGE

Q NO	ITEMS	RESPONSES (MAKE SURE YOU FILL ALL COLUMNS)				
014	IF YES, what is the type of health professional education, the duration, status, year of completion and evidence?					
		a) Type of education	b) Duration of Training? (years)	c) Is education completed?	d) Year of completion	(e) Evidence of Certification
Line No.	Education Program (No. of years of training)	Yes No		Yes No Still on	DK=Don't Know	Yes No
1	Community Health Officer (2)	1 2		1 2 3		1 2
2	Pharmacy Technician (2)	1 2		1 2 3		1 2
3	Auxiliary Nurse (2)	1 2		1 2 3		1 2
4	Junior Community Health Extension Worker (2)	1 2		1 2 3		1 2
5	Senior Community Health Extension Worker (2)	1 2		1 2 3		1 2
6	General Nursing (3)	1 2		1 2 3		1 2
7	Nurse-Midwife (4)	1 2		1 2 3		1 2
8	Environmental Health Officer (3)	1 2		1 2 3		1 2
9	Other, specify: ________________	1 2		1 2 3		1 2

Q NO	ITEMS	RESPONSES
015	Have you been ever trained as a role model mother or a voluntary health care worker?	ROLE MODEL MOTHER…………………………..1<br>OTHER VOLUNTARY HEALTH CARE WORKER ________________………………......2<br>(SPECIFY)<br>NO OTHER HEALTH WORKER TRAINING…..3<br>**IF NO OTHER TRAINING, GO TO Q 018**

CONTINUE ON NEXT PAGE

Q NO	ITEMS	RESPONSES (MAKE SURE YOU FILL ALL COLUMNS)
016	IF YES, Duration of training?<br>**IF LESS THAN 1 YEAR WRITE 00 IN YEARS**<br>**IF LESS THAN 1 MONTH WRITE 00 IN MONTHS AND YEARS**	☐☐ YEARS<br>☐☐ MONTHS<br>☐☐ WEEKS<br>DON'T KNOW/DON'T REMEMBER…..88
017	IF YES, Topics of training?<br>**READ ALL RESPONSES**<br>**CIRCLE ALL THAT APPLY**	MALARIA .... 1<br>DIARRHEA... 2<br>ACUTE RESPIRATORY INFECTION... 3<br>OTHER 1_________________ .... 4<br>(SPECIFY)<br>OTHER 2_________________ .... 5<br>(SPECIFY)
018	How many PMV shops do you have?	☐☐
020	Is PMV work your primary occupation/employment?	YES…………………………………..1 **GO TO Interview**<br>NO……………………………………2
021	IF NO,<br>What is your primary occupation/employment?	

Q NO	ITEMS		Total	# of Apprentices
019	FOR EACH SHOP UP TO 4 SHOPS,<br>Excluding yourself, how many other people work in each shop (including regular paid employees, apprentices, and non-paid family members/volunteers)?<br>How many apprentices?			
		Shop 1		
		Shop 2		
		Shop 3		
		Shop 4		

Staff Interview

1. How long have you occupied the current position as an executive of the association? ***Ask for previous positions occupied if any.***

2. What are the specific duties of an officer occupying your position?
    - ***For Financial Secretary*, *probe for details of financial management system***
        - Membership dues, special levies etc
        - Ownership of association bank account, signatories to the account
        - Activities for which the money collected is utilized
    - ***For Head of PMV Task Force where such exists, probe for***
        - How many members are in the task force and what are their individual roles?
        - How are members of the task force selected?
        - What are the specific functions of the task force?
        - How does the task force organize its activities?
        - How do they enforce compliance and sanction to the erring members?
        - How does the task force collaborate with the statutory regulating agencies to monitor their members?
    - ***For Public Relations Officer, where such exists, ask***
        - How does information get across to members?
        - How are members mobilized for group activities e.g. meeting, training etc.?
        - How does the association interact with regulatory agencies?
    - ***For Welfare Officer, where such exists, ask***
        - What activities do you carry out to improve the welfare of members?
        - How do you carry out these activities? Who works with you?
        - If they give money to members, how much do you give to members vs. executive members?
        - What effect do you think the welfare activities have on members?
        - How do the welfare activities affect the association?
    - ***For Chairman, where such exists, ask***
        - Planning? Coordination of members?

3. Apart from these specific duties, what other duties do you/are you expected to perform?

4. What are the challenges you face in performing your role?

5. How can the performance of the PMV Association be improved? How could your role change to enhance the performance of the PMV Association?

Improving malaria treatment in the LGA

6. What do the anti-malaria treatment guidelines say:

    a) About diagnosing malaria

    b) About treating malaria, including uncomplicated vs. severe malaria

    c) About the role PMVs should play in diagnosing and treating malaria?

    If they are not aware of the guidelines, inform them. Show a copy of the guidelines.

7. The government anti-malaria treatment guidelines recommend that all malaria suspects be tested for malaria before being prescribed treatment. New rapid diagnostic tests for malaria exist which allow trained lay workers to conduct this testing.

    Mention that it involves taking blood then show them the test and list the steps in demonstration

    a. What barriers would PMVs have in using these rapid diagnostic tests with patients?

    b. What challenges would PMVs face in encouraging clients to purchase RDTs?

    c. What barriers would PMVs associations have in encouraging PMVs to use of these rapid diagnostic tests with patients?

    d. What type of incentives could the PMV associations propose to make PMVs conduct rapid diagnostic tests for all suspected malaria cases before treatment?

8. ACTs are the government recommended first line medicines for treating malaria patients.

    a. What challenges would PMVs face in encouraging clients to choose ACTs over other anti-malarial drugs?

    b. What barriers would PMVs associations have in encouraging PMVs to recommended ACTs as first line treatment for malaria for their patients?

    c. What type of incentives would the PMV associations propose to make PMVs prescribe ACTs as first line treatment for malaria?

9. What role do you think you can play in improving the malaria diagnosis and treatment practices of your members?

10. What can be done to help you to better perform your role as an executive member of your LGA PMV Association?

11. What role do you think your PMV association could play in making your members (PMVs) committed to conducting rapid diagnostic tests for all suspected malaria cases before treatment and (b) prescribing ACTs as first line treatment for malaria in line with the NMCP 2011 guidelines?

We have come to the end of the interview. Thank you for your time used in talking to us.
