# Supplementary material for: The potential role of patent and proprietary medicine vendors’ associations in improving the quality of services in Nigeria’s drug shops
Source: BMC Health Serv Res. 2020 Jun 22;20:567. doi: 10.1186/s12913-020-05379-z (PMC7310190; doi:10.1186/s12913-020-05379-z)
Supplement: Supplementary file 4 — Additional file 4. Regulator Key Informant Interview. This is the guide used to interview representatives of regulatory agencies about the specific role of their organization in regulating the activities of PPMVs. [file 12913_2020_5379_MOESM4_ESM.pdf]

Informed Consent for Key Informant Interviews with Government Regulators

THIS COPY TO BE GIVEN TO INTERVIEWEE

This project we want you to join is a study. The University of Ibadan (UI) Future Health System Research Group and the Niger Delta University in Nigeria in collaboration with the West African Infectious Disease Institute are carrying out a study "Strengthening Patent Medicine Vendors' Associations in Nigeria for Improved Malaria Management (SPANIMM)" which involves collecting information from government regulators through interview on their experiences working with the PMV associations.

If you choose to participate in this study, we will ask you questions about your personal experiences working with PMV associations. We will also ask about what types of services that your organization has been providing to PMV associations, and the challenges faced in performing regulatory functions. We will explore your attitude to the strengthening of PMV Associations capacity for enhanced role in improving PMVs malaria management practices. We do not require your names for this survey interview. We will talk to different government health regulators at National and local levels.

The questions are general but if you find that some questions are not going well with you, please do not feel compelled to answer any of them for any reason. We will talk to you for about 45 -60 minutes. You can decide if you want to take part in this interview survey. Taking part in this study will not cost you or your family anything. You may also leave the survey interview at any time. You can leave for any reason without any problems. You and your family may not get any direct benefits from being in this study. What you tell us will help us better understand partnership issues and assist in developing a pilot intervention(s) for PMV associations to enhance PMVs diagnosis and treatment of malaria in line with the revised 2011 National Policy on Diagnosis and Treatment of Malaria. There are no risks involved in your participation in this interview. The benefit of this study to you is that you will have an opportunity to talk about your experiences and be heard as well as have the opportunity to participate in health related decisions concerning malaria management in rural areas. As a result of this study, PMVs may be able to diagnose and treat malaria more effectively in rural areas thereby reducing malaria illness and deaths.

We will provide you with a present as a token of our appreciation of your time and effort for participating in this interview. You would not incur any financial costs for being in this study. The only cost of your participation is the time you allow for answering the interview questions. Your name and what you say to us for this study will be kept private as much as the law allows.

Do you have any questions about the study?

If you have any questions about your rights in the study or in case of emergency, you may contact the following persons during the study and in the future:

- Professor Oladimeji Oladepo at the Dept. of Health Promotion & Education, Faculty of Public Health, College of Medicine, University of Ibadan, Ibadan, Oyo State, Nigeria, Phone:0803-326-3302
- Dr. Abisoye Oyeyemi, Department of Community Medicine, Faculty of Clinical Sciences, College of Health Sciences, Niger Delta University Wilberforce Island, Bayelsa State in Nigeria, Phone: 0803-704-9837
- Ms. Sarah Burnett at Accordia Global Health Foundation,1101 14th Street NW, Suite 801, Washington, DC 20005, phone: + 1 202 534 1200

If you agree to participate in this interview you can say that you agree and we will record your response on the tape recorder.

Tool 7

Regulator Key Informant Interview

Introduction

My name is ______________________________and my companion is________________. We work for a research group based in the Niger Delta University. We are doing a project funded by the West African Infectious Diseases Institute. We want to ask you a few questions on issues relating to the Patent Medicine Vendors. This interview is important to help us understand the role of your organization as a regulatory body and your experience working with the Patent Medicine Vendors. Your answers to all the questions we ask will be kept secret and we shall be grateful if you are honest in answering all the questions. If you agree, we would like to capture this interview on a tape recorder to ensure that we do not miss any part of the interview. Thank you.

Note takers **must** record the following in notes:

1. Date of Interview
2. Start time of interview
3. Stop time of interview
4. Interviewer Name
5. Note taker Name
6. Interview Tool used
7. Organization of Interviewee
8. Gender of Interviewee

Section1. Demographic Information

1. What is your title/designation?
2. What is your highest level of education and degree/certificate?
3. How long have you been in this organization?
4. How long have you occupied the current position? ***Ask for previous positions occupied in the organization if any.***
5. What are the statutory functions of your organization?

Section 2. Interaction with PMVs

6. What kind of relationship exists between your organization and the PMV association? How do you work with the PMV Association and PMVs? Probe:
    a. meet with them at scheduled intervals,
    b. organize/support training,
    c. regulatory relationship

7. What specific roles does your organization have in regulating PMV activities?
    a. Registration, monitoring, training, any other roles?
    b. How does the organization perform these roles?
    c. Any special unit dedicated to this?
    d. Monitoring, ***Probes:***
        i. How often scheduled for? How often does it occur?
        ii. Who does it?
        iii. What do they check for during monitoring (describe process)?
        iv. What happens if a PMV does/does not meet standards?
        v. How effective do you feel the monitoring is? Why or why not?
        vi. What would make it more effective?
    e. Registration/Licensing –***If PMVs register or get licensed by the organization, ask for the process of registration/licensing:***
        i. What requirements must a PMV meet in order to register/get licensed with your organization?
        ii. What requirements must a PMV meet in order to maintain their registration/license status with your organization?
        iii. What are the requirements for opening a PMV shop?
        iv. Average time between applying and award of license,
        v. Registration/licensing fee,
        vi. Fee for renewal of practicing license,
    f. Any other fees paid periodically from state/LGA PMV Association or individual PMVs?
        i. ***Probe for*** variation probably based on the membership strength of the PMV association or other parameters

8. Does your organization have powers to impose sanctions on erring PMV Association members? What kind of sanctions can be imposed? What effect do you think these sanctions have?

9. What has been the experience of your organization in working with PMV associations? With PMVs themselves?

10. What challenges does your organization face in performing your role in regulating the activities of the PMVs?
11. How can PMV associations be strengthened to help your organization meet your goals?

12. Are you aware of the 2011National Policy on Diagnosis and Treatment of Malaria? What do the anti-malaria treatment guidelines say about the role of PMVs in diagnosing and treating malaria, including uncomplicated and severe malaria?

If they don't know the 2011 National Policy on Diagnosis and Treatment of Malaria, explain it now and show them the copy.

13. According to the2011 National Policy on Diagnosis and Treatment of Malaria, PMVs are expected to have the following role in malaria prevention, diagnosis and treatment:
    a. To stock and sell insecticide treated bed nets (ITNs) to prevent malaria.
    b. To stock and provide rapid diagnostics tests for malaria prior to treatment of uncomplicated malaria and referral of suspected severe malaria cases. ***Demonstrate how RDTs work - that it involves taking blood then show them the test and list the steps in demonstration***
    c. To provide Artemisinin-based combination therapy (ACT) for the treatment of confirmed malaria cases.

    What are your opinions about this expected role of PMVs in the prevention and treatment of malaria? ***Go over each guideline separately in probing***

14. How could your organization better support PMV Associations in ensuring that the NMCP 2011 guidelines are carried out by PMVs?
    a. To ensure that PMVs regularly conduct rapid diagnostic testing for all malaria suspects
    b. To ensure PMVs prescribe ACTs as first line malaria treatment

15. Is there anything else you'd like to tell us?

We have come to the end of the interview. Thank you for your time used in talking to us.
